# Characterization of RON protein isoforms in pancreatic cancer: implications for biology and therapeutics

**DOI:** 10.18632/oncotarget.10009

**Published:** 2016-06-14

**Authors:** Jeffery Chakedis, Randall French, Michele Babicky, Dawn Jaquish, Evangeline Mose, Peter Cheng, Patrick Holman, Haleigh Howard, Jaclyn Miyamoto, Paula Porras, Zakk Walterscheid, Carsten Schultz-Fademrecht, Christina Esdar, Oliver Schadt, Jan Eickhoff, Andrew M. Lowy

**Affiliations:** ^1^ Department of Surgery, Division of Surgical Oncology, Moores Cancer Center, University of California, San Diego, La Jolla, CA, USA; ^2^ Lead Discovery Center GmbH, Dortmund, Germany; ^3^ Merck KGaA, Darmstadt, Germany

**Keywords:** RON, pancreatic cancer, isoform, alternative splicing, tyrosine kinase inhibitor

## Abstract

The RON tyrosine kinase receptor is under investigation as a novel target in pancreatic cancer. While RON mutations are uncommon, RON isoforms are produced in cancer cells via a variety of mechanisms. In this study we sought to: 1) characterize RON isoform expression in pancreatic cancer, 2) investigate mechanisms that regulate isoform expression, and 3) determine how various isoforms effect gene expression, oncogenic phenotypes and responses to RON directed therapies. We quantified RON transcripts in human pancreatic cancer and found expression levels 2500 fold that of normal pancreas with RON isoform expression comprising nearly 50% of total transcript. RNA seq studies revealed that the short form (sfRON) and P5P6 isoforms which have ligand independent activity, induce markedly different patterns of gene expression than wild type RON. We found that transcription of RON isoforms is regulated by promoter hypermethylation as the DNA demethylating agent 5-aza-2′-deoxycytidine decreased all RON transcripts in a subset of pancreatic cancer cell lines. The viability of sfRON-expressing HPDE cells was reduced by a RON specific small molecule inhibitor, while a therapeutic monoclonal antibody had no demonstrable effects. In summary, RON isoforms may comprise half of total RON transcript in human pancreatic cancer and their expression is regulated at least in part by promoter hypermethylation. RON isoforms activate distinct patterns of gene expression, have transforming activity and differential responses to RON directed therapies. These findings further our understanding of RON biology in pancreatic cancer and have implications for therapeutic strategies to target RON activity.

## INTRODUCTION

Pancreatic cancer is a disease of aggressive biology marked by late stage presentation, and a 6% 5 year survival rate. With no effective means of early detection and only weakly active therapeutic options, the pancreatic cancer death rate is expected to increase over the next 20 years to become the second leading cause of cancer-related death [1]. The development of new and more effective therapies is therefore a huge unmet need. RON is a tyrosine kinase receptor oncogene aberrantly expressed in pancreatic cancer as well as other epithelial malignancies (breast, prostate, colon, lung) [2]. Overexpression of the gene for RON, *MST1R*, is a signature of KRAS mutant cells, some of which are dependent on RON expression for viability [3]. RON belongs to the c-MET kinase family, however unlike MET, RON is infrequently activated through by gain of function mutations. Instead RON is activated in various ways including: 1) binding of its cognate ligand Macrophage Stimulating Protein (MSP), 2) overexpression resulting in dimerization and autophosphorylation, 3) expression of constitutively active variants produced via alternative splicing or transcription. Targeting RON as a putative therapeutic strategy is supported by prior observations that cancer cells which overexpress the protein are dependent on signaling for growth, survival, angiogenesis, and motility [4]. Blocking RON activity has been shown *in vitro* to decrease cell invasion, sensitize cells to chemotherapy, and decrease the *in vivo* growth of tumor xenografts [5–7].

The concept of a gene is being redefined as we now know that 90% of coding genes undergo alternative splicing to produce proteins with altered activities [8]. Data from the ENCODE project shows that isoform production plateaus at 10-12 isoforms per gene and that at this expression level, the wild type protein represents only 50% of the total transcripts [9]. Alternative splicing has been evolutionarily conserved as a function to enhance protein diversity with limited number of genetic material [10]. In total, nine protein isoforms of RON have been reported in the literature. Most commonly, RNA transcripts are alternatively spliced to produce proteins that have skipping of exons or inclusion of introns. Many of these isoforms such as RONΔ55 also known as short form (sfRON), RONΔ165, RONΔ160 and RON P5P6 are constitutively phosphorylated when expressed and contribute to oncogenic phenotypes [11–14]. SfRON is created by an alternative transcription start in exon 11 that omits the N-terminus while retaining the intracellular kinase domain [15]. SfRON is over-expressed in breast cancer and induces cellular invasion, epithelial to mesenchymal transition, and metastasis *in vivo*, yet the frequency of its expression in pancreatic cancer has not been reported [16].

RON directed therapeutics are now being investigated in early phase clinical trials. RON8 (Narnatumab, ImClone) is a monoclonal antibody which is highly specific for RON and blocks ligand binding to reduce tumor xenograft growth [7]. Multiple RON/c-MET small molecule tyrosine kinase inhibitors similarly exhibit pre-clinical efficacy [17, 18]. These small molecule inhibitors are not specific for RON and always inhibit a wide variety of kinase targets with off target effects unrelated to RON inhibition [19]. Epigenetic therapies may offer another strategy to target RON. Analysis of genomic DNA methylation patterns found that in pancreatic and breast cancer, decreasing RON promoter methylation increases transcript expression and the converse [20, 21]. Mechanistically, Full length RON (flRON) and sfRON expression is regulated by the methylation status of 2 separate promoters upstream of the *MST1R* gene [22]. Combination RON specific and epigenetic therapies may also be an effective strategy as RON8 treatment sensitized pancreatic cancer cell lines to histone deacetylase inhibitors [23].

Ultimately RON is a promising therapeutic target with several agents currently in early phase clinical trials and new inhibitors in development. RON isoforms may also be therapeutic targets as their expression could subvert any benefit derived from inhibiting the full length protein. In this study we sought to: 1) characterize patterns of RON isoform expression in pancreatic cancer, 2) understand their effects on patterns of genome wide transcription, 3) investigate how they may respond to RON therapeutics. Such information will be necessary to properly develop and interrogate the efficacy of RON targeted therapies in cancers which are known to express RON isoforms.

## RESULTS

### Repertoire of RON isoform expression in pancreatic cancer

**Figure 1:**
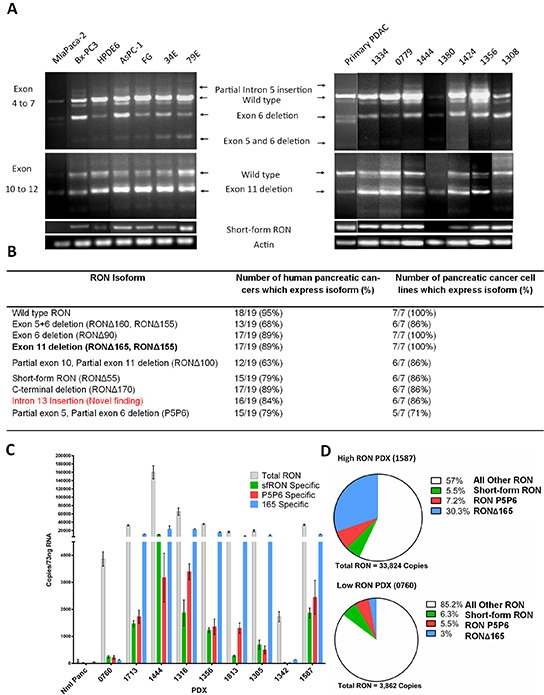
RON isoforms are highly expressed in human pancreatic cancer specimens **A.** End point PCR of pancreatic cancer cell lines, human pancreatic ductal adenocarcinoma (PDAC), and patient derived xenografts show the products of alternative splicing. Primers flanking regions which are highly spliced and primers specific to the short form were used to resolve RNA isoforms. **B.** The PCR data summarized for all cell lines and patient derived xenografts. Exon 11 skipping is the most common event and 2 new splice products were discovered – intron 13 insertion and partial 5 and 6 skipping. **C.** Absolute quantification PCR quantifies RON expression in patient derived xenografts. As RON expression increases there is a corresponding increase in isoform transcript number. **D.** The percentage RONΔ165 transcripts increased as RON transcripts increased while the percentages of short form RON and P5P6 were similar. Overall in high RON expressing PDXs almost 50% of transcripts can be attributed to 3 of the isoforms. All other RON transcripts corresponds to the total number of RON transcripts minus the combined measured isoform transcripts. This includes the full length and all other not measured isoforms.

The spectrum of RON isoform expression has not been comprehensively examined in pancreatic cancer. In order to test our hypothesis that these isoforms are expressed in pancreatic cancer and may contribute to its aggressive phenotype, we first characterized which isoforms are expressed in a panel of pancreatic cancer cell lines and low passage patient derived xenografts. We began by using RT-PCR to examine exons 4 to 7 and exons 10 to 12 of RON pre-mRNA that are highly spliced (Figure 1A) [13, 24]. Bands were sequenced and determined to be specific for splice products previously described by our group and others, as well as a newly identified intron 13 insertion isoform. The discovery of intron of the 13 insertion was proved by using primers that flank exons 10-14 and sequencing the PCR products (Supplementary Figure S1). Primers specific for sfRON, RONΔ170, RONΔ165, Intron 13 insertion and P5P6 were constructed (Supplementary Table S1) based on unique splice junctions and used to determine their specific expression in each human PDX and pancreatic cancer cell line. This analysis (Figure 1B) revealed that full length RON transcripts are present in 95% of PDXs and the exon 11 skipping isoform is the most commonly found splicing event.

We next sought to quantitate isoform expression as a potential indication of their biologic relevance. There are no antibodies specific for any RON isoform and their similar molecular weights makes it difficult to resolve isoforms from the full length protein. We therefore used absolute quantitative PCR to determine the transcript numbers of the full length RON transcript and 3 specific isoforms using the specific primers stated in Supplementary Table S1 (Figure 1C). We derived isoform specific transcript numbers from normal pancreas and a representative sample of PDXs using standard curves for each transcript. Compared to normal pancreas, we found that total RON transcript expression ranges from 30-60 fold increased in what we have termed “low RON expressing PDXs” and 500-2500 fold increased in “high RON expressing PDXs”. As total RON transcript expression increases, we observed a concomitant increase in RON isoform transcription as well. Comparing low RON to high RON expressing PDXs (Figure 1D) there are similar percentages of sfRON and P5P6, however the expression of RONΔ165 increases up to 30%. These three isoforms make up 43% of RON transcripts expressed in high RON PDXs. Overall this data confirms that RON isoforms are expressed in pancreatic cancer and their abundance suggests the potential for biological impact.

### DNA demethylation modulates RON expression in pancreatic cancer

**Figure 2:**
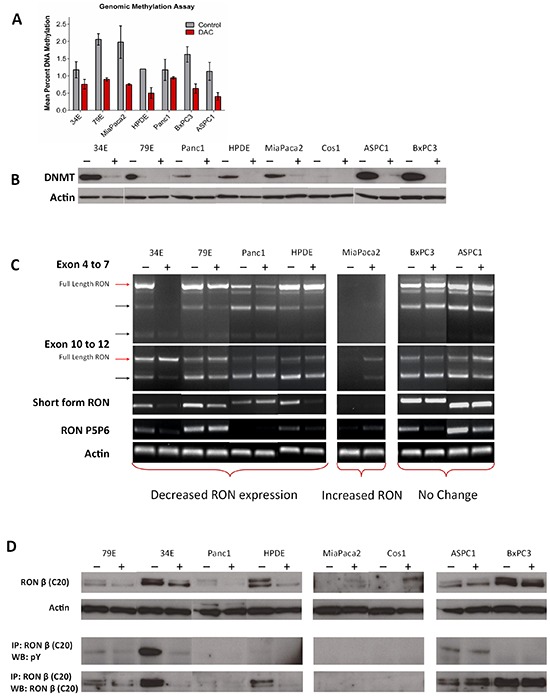
DNA demethylating agents modulate RON isoform expression **A.** Pancreatic cancer cell lines were treated with Decitabine (DAC, 4μM) for 96 hours and assessed for percent genomic DNA methylation. **B.** The treatment was effective at decreasing DNA methylation and depleting its target – DNA methyltransferase 1 (DNMT1) in all cell lines. **C.** PCR showed that in a subset of cell lines which normally express RON (34E, 79E, Panc1, HPDE) DAC treatment decreased flRON expression and isoform expression. In cell lines which do not express RON (MiaPaca2) DAC treatment induces its expression. **D.** The PCR data is confirmed using western blot with the same trends of up and downregulation seen in concordant cell lines. The western blots in this image derive from different gels.

After demonstrating the profound overexpression of RON isoforms in pancreatic cancer, we sought to explore the influence of DNA methylation on isoform expression. It has been shown previously that full length RON expression levels are related to the methylation status of its promoter and HDAC inhibitors can reduce full length RON expression in pancreatic cancer cell lines [23]. We sought to investigate whether demethylation by another epigenetic modifying agent, 5-aza-2′-deoxycytidine (Decitabine, DAC), could modulate expression of full length RON and its isoforms in pancreatic cancer cells. Baseline genomic methylation analysis (Figure 2A) of pancreatic cancer cell lines revealed that relative to HPDE cells (1.2% methylation) some lines were hypomethylated (ASPC1-1.13%, 34E-1.17%, Panc1-1.17%) while others were hypermethylated (BxPC3-1.6%, MiaPaca2-1.98%, 79E-2.06%). After exposure to DAC for 96 hours at a moderate dose (4 μM), genomic methylation was decreased below normal levels (0.4%-0.9%) in all cell lines and DNA MethylTransferase 1 (DNMT1) protein levels were depleted (Figure 2B). We next assessed RON transcript level using PCR (Figure 2C) and found the effects of demethylating therapy on RON expression differed depending on the cell line. We noted decreased levels of full length RON transcripts in the 79E, 34E, Panc1, and HPDE cell lines and increased levels in MiaPaca2. MiaPaca2 does not normally express RON, but demethylation induced RON expression. Transcripts specific for sfRON decreased in 79E, 34E, and HPDE cell lines but did not change in the others. Expression of other RON isoforms decreased in 34E, 79E, ASPC1, and BxPC3 lines, with less exon 11 skipping and partial skipping of exon 5 and 6.

We next examined the corresponding protein expression (Figure 2D) and found concordance with our RNA data. RON protein expression decreased in DAC treated 79E, 34E, Panc1, and HPDE cell lines. There was a similar induction of RON expression in MP2 and Cos1, neither of which typically express RON. The decrease in total RON protein expression resulted in a corresponding decrease in the amount of constitutively active RON present. In contrast, induction of RON expression in MP2 did not result in constitutively active protein. Given the multiplicity of DAC effects, we cannot be certain that the changes we observed are direct effects on RON promoter methylation or secondary to effects on other transcription factors that regulate RON transcription. Ultimately however, our results clearly demonstrate that in pancreatic cancer cells, expression of RON and its isoforms are regulated by DNA methylation and that demethylating agents can modulate RON expression. Further, the effects of DNA methylation relate to the basal methylation status of the particular cell line.

### Short form RON induces oncogenic phenotypes and has transforming capacity

**Figure 3:**
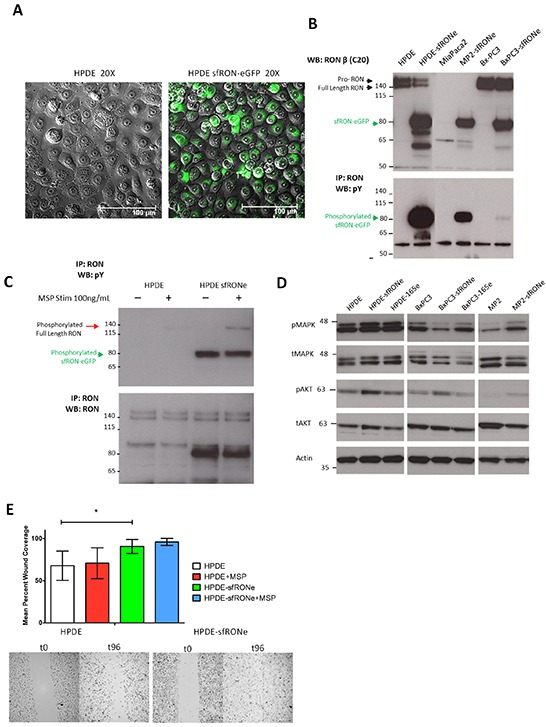
Short form RON induces oncogenic phenotypes and has transforming capacity **A.** HPDE cell line expressing the sfRON-eGFP fusion protein showed no change in cell morphology but expressed high levels of the protein. **B.** The sfRONe protein is expressed in 3 different cell lines at similar levels. The protein is constitutively active in each cell line but with different levels of phosphorylation. **C.** MSP ligand activates full length RON but not short form RON. **D.** Signaling downstream of sfRON activates the MAPK and AKT pathway. **E.** Short form RON expression in the HPDE cell line increases cell motility more than full length RON activation with MSP alone.

Previous studies suggest that RON isoforms may promote an aggressive phenotype that is distinct from that of the full length protein [14, 16]. Therefore, we sought to determine the biologic activity of several RON isoforms *in vitro* and *in vivo*. For these studies, we established cell lines stably expressing RON isoforms both with tagged with enhanced green fluorescent protein (eGFP) and without. We found that signaling and expression in the eGFP and non-tagged versions was identical [14]. The parental cell lines utilized are an immortalized human pancreatic ductal epithelial cells (HPDE, Figure 3A), a RON expressing pancreatic cancer cell line in which full length RON is active (Bx-PC3), and a pancreatic cancer cell line that does not express RON (MiaPaca2). Western blot of the 3 cell lines expressing sfRON (Figure 3B) shows that the protein is expressed in similar quantities in each cell line, but the constitutive phosphorylation pattern differs. Full length RON was not phosphorylated in the presence of sfRON in the HPDE cell line and sfRON did not respond to MSP stimulation, an observation that has been previously reported in other cell lines (Figure 3C) [15]. RONΔ165 expression did not change cell morphology but also resulted in a constitutively active protein in 2 different cell lines (Supplementary Figure S3A and S3B).

We next examined the effects of isoform expression on canonical downstream signaling pathways and found that in keeping with previous reports in breast cancer, sfRON expression increased phosphorylation of AKT in all 3 cell lines (Figure 3D) [16]. ERK phosphorylation was increased in HPDE and MP2 but not BxPC3. In contrast, RONΔ165, another constitutively active isoform, induced increases in MAPK phosphorylation only in the HPDE cell line. Consistent with other studies, we failed to observe any changes in proliferation secondary to overexpression of any RON isoform, the changes in downstream signaling we observed did result in other functional differences in cells expressing sfRON (Supplementary Figure S3C) [16]. HPDE cells expressing sfRON demonstrated increased migration as measured by a scratch wound assay at 96 hours (p = 0.011, Figure 3E). MSP stimulation did not significantly increase cell migration in parental HPDE or HPDE sfRON cell lines. As we have previously shown that RON signaling uses the MAPK pathway to increase VEGF secretion by pancreatic cancer cells [4], we assessed RON isoform mediated changes in VEGF secretion (Supplementary Figure S4A). We found a cell context dependent effect as sfRON tripled VEGF expression in the HPDE cell line (654 pg/mL vs 193 pg/mL, p < 0.001) but did not change expression in Bx-PC3 (1114 vs 1086 pg/mL) and expression in MiaPaca2 was mildly decreased (216 vs 378 pg/mL). Finally, we performed a colony forming assay in NIH-3T3 cells transfected with sfRON. We found that sfRON expression resulted in significantly higher colony formation (p-value <0.001) than the vector alone, but the effect was not as potent as expression of mutant KRAS (Supplementary Figure S4B). Finally we examined signaling through other tyrosine kinases and found that sfRON expression increased phosphorylation of MET but not IGF1R or EGFR (Supplementary Figure S4C). EGFR total expression was greatly reduced following sfRON expression but EGFR phosphorylation was unchanged. Overall these experiments demonstrate that sfRON expression in pancreatic ductal epithelial cells results in enhanced cell migration and VEGF secretion as well as oncogenic transformation.

### Short form RON expression in human pancreatic ductal epithelial cells is tumorigenic

**Figure 4:**
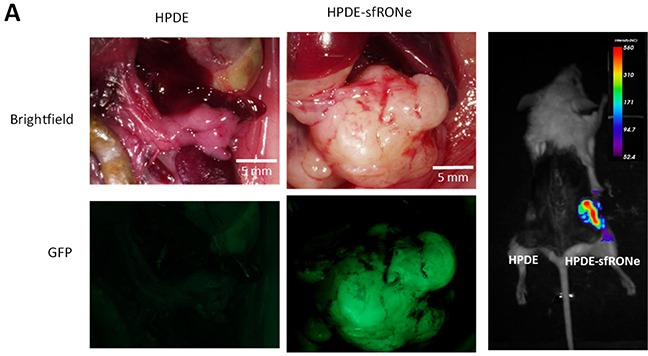
*In vivo* expression of short form RON is tumorigenic **A.** Orthotopic injection of HPDE sfRONe cells into pancreata of NSG produced tumors while injection of the parental HPDE line did not. Tumors formed in both orthotopic and subcutaneous injections.

Given that expression of sfRON induces oncogenic phenotypes *in vitro*, we sought to determine if these phenotypes were relevant to growth *in vivo*. For *in vivo* experiments, we utilized an orthotopic cell injection model. The HPDE cell line has been previously shown to be non-tumorigenic in immunodeficient mice [14, 25]. We injected one million HPDE or HPDE-sfRONe cells into the pancreata of NOD-SCID Gamma (NSG) mice as well as subcutaneously. We found that after 12 weeks, tumors formed in the sfRONe group (Figure 4A) but not in the parental line. The tumors are fluorescent as seen in the gross images and are thus detectable with GFP imaging.

Expression of sfRON and RONΔ165 has been reported to induce an epithelial-mesenchymal shift in breast cancer cells [16]. As such we sought to examine the effects of isoform expression on epithelial (E-cadherin) and mesenchymal (vimentin and alpha smooth muscle actin) marker protein expression in HPDE cells. We found no evidence of an epithelial-mesenchymal shift in cells expressing sfRON, either by morphology or marker expression (Supplementary Figure S5A). Interestingly, immunostaining for the same markers in HPDE-sfRONe tumors showed that E-cadherin expression and cell morphology was variable throughout the tumor (Supplementary Figure S5B). Areas of high E-cadherin expression correlated with round cell morphology. However in adjacent areas of the tumor there were areas of loss of E-cadherin associated with an increase in mesenchymal morphology. This indicates that in tumors transformed by sfRON, interactions with the surrounding tumor microenvironment may be necessary to modulate epithelial-mesenchymal transition.

### Monoclonal antibody therapy does not inhibit constitutive isoform activation

**Figure 5:**
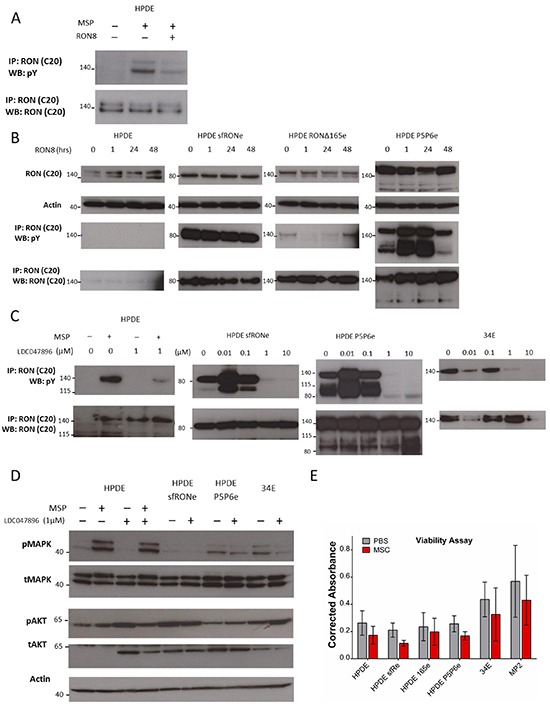
A RON specific small molecule inhibitor blocks isoform constitutive activation while monoclonal antibody therapy does not **A.** RON8 is a monoclonal antibody directed at the ligand binding extracellular domain of RON which blocks MSP induced phosphorylation in the HPDE cells line. **B.** Isoform expressing cell lines were not inhibited by RON8. RONΔ165 phosphorylation was decreased up to 24 hours of treatment but then returned to baseline levels. **C.** LDC047896 treatment for 24 hours at 1 μM prior to stimulation with RON ligand MSP blocked full length RON phosphorylation. Constitutive activation of sfRON and P5P6 was removed at a dose of 1 μM. Similarly, constitutive activation of full length RON in the 34E cell line was removed at a 1 μM dose. **D.** Activation of flRON was blocked at a 1 μM concentration after stimulation with MSP, but it did not block MAPK phosphorylation. MAPK signaling in the isoform expressing and 34E cell lines was decreased at a dose of 1 μM but did not decrease AKT signaling. D, Viability of HPDE sfRON and P5P6 was decreased significantly (p 0.001 and 0f.007) but not in other lines. The western blots in this image were derived from different gels.

While we and others have demonstrated that RON isoforms promote oncogenesis, the effects of RON directed therapies against RON isoforms has not been previously reported. Given that these isoforms may represent close to 50% of the RON transcripts expressed, their response to RON directed therapies clearly must be evaluated. We first re-affirmed that the RON monoclonal antibody, RON8, is able to bind to the full length receptor and block MSP induced phosphorylation (Figure 5A). We then treated HPDE isoform expressing cells with RON8 for 1 to 48 hours (Figure 5B). We found that total RON and RON isoform protein levels were stable with RON8 treatment. RON8 binds to the SEMA domain of full length RON and does not possess agonistic binding activity. As sfRON does not possess a Sema domain, not surprisingly its phosphorylation was not inhibited by RON8. Similarly RON P5P6 phosphorylation was unaffected by RON8. Unexpectedly, RONΔ165 phosphorylation declined with RON8 treatment and then returned by 48 hours, likely due to a compensatory mechanism. This is interesting as RON Δ165 is not cleaved into α and β subunits and is reported to form intracellular oligomers instead of trafficking to the plasma membrane [12]. Overall this data shows that the RON8 monoclonal antibody, though effective at blocking MSP induced phosphorylation of the full-length protein, it does not impact the phosphorylation status of constitutively active isoforms when expressed in HPDE cells. This provides further evidence that isoform activation is induced by ligand independent mechanisms and suggest that monoclonal antibody therapy targeting ligand binding may have therapeutic limitations in tumors expressing significant amounts of specific RON isoforms.

### A RON specific small molecule inhibitor removes constitutive isoform activation

Small molecule kinase inhibitors can act by competitively inhibiting ATP binding within the kinase domain. Most of the publications examining the biological role of RON activity with small molecule inhibitors have taken advantage of the small molecule inhibitor BMS-777607, which is currently in phase 1 clinical trials [17, 19]. However conclusions drawn from studies with this molecule have to be taken with caution as BMS-777607 is not selective for RON and inhibits the closely related kinase cMet on a biochemical and cellular level. As RON and cMET interact with each other, and both promote tumor growth and invasion, highly selective inhibitors are need to elucidate the function of RON kinase activity in cancer [26]. Therefore, we used the novel highly selective RON kinase inhibitor LDC047896, a small molecule in pre-clinical development, to examine the role of RON isoform kinase activity in pancreatic cancer. In contrast to BMS-777607, LDC047896 is highly specific for RON on a biochemical level, and even more on a cellular level (Table 1). In addition, broader kinase profiling revealed a high level of selectivity of this compound in a panel of 240 different kinases (Supplementary Table S2).

**Table 1:** Selectivity of LDC047896 for RON on biochemical and cellular levels in comparison to BMS777607

	Biochemical RON kinase assay IC_50_[nM]	Biochemical cMET kinase assay IC_50_[nM]	Cellular RON inhibition IC_50_[nM]	Cellular cMET inhibition IC_50_[nM]
**LDC047896**	21	74	1.6	2000
**BMS777607**	12	1.4	14	150

We treated HPDE parental lines and found that LDC047896 inhibited full length RON phosphorylation in the setting of MSP stimulation at 1μM (Figure 5C). Similarly, constitutive activity of sfRON and RON P5P6 is inhibited at the same concentration. A primary human pancreatic cancer cell line (34E) which has constitutive full length RON activation, was also inhibited at the same concentration. We therefore investigated the response of canonical AKT and MAPK kinase signaling pathways after treatment with LDC047896 (Figure 5D). In HPDE, MSP stimulation induces MAPK activation; although LDC047896 blocks MSP induced RON phosphorylation it does not block MAPK phosphorylation. This suggests that MSP binding to flRON induces MAPK through a mechanism other than RON kinase phosphorylation or more likely, that HPDE cells can circumvent RON inhibition by increasing MAPK through an alternate signaling pathway. For isoform expressing HPDE lines (sfRONe and P5P6) and 34E, LDC047896 decreases MAPK activation at 1μM. In contrast there was no change in AKT signaling after inhibition with LDC047896, again suggesting that cells may find other means to activate this pathway in the setting of RON kinase blockade. We tested cell viability following RON kinase inhibition using an MTT assay after 48 hours of treatment with LDC047896 at 1 μM (Figure 5E). Cell viability was markedly reduced in isoform expressing cell lines HPDE sfRONe (p< 0.001) and HPDE P5P6e (p< 0.007) but not in HPDE parental, MP2, or 34E cells (p 0.08, 0.34, 0.29 respectively). This demonstrates that LDC047896 had a specific effect on isoform expressing cell lines and that the effect on viability is therefore not likely an off target effect.

### RON isoform expression separate and distinct effects on the cell transcriptome

**Figure 6:**
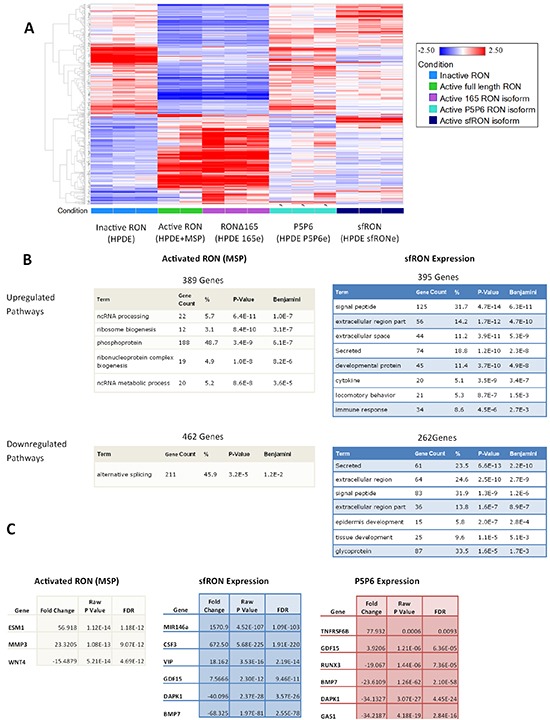
RNA-seq of RON isoform expressing cell lines show differential gene expression **A.** Heatmap of gene expression patterns comparing each cell line. **B.** DAVID gene ontology pathways which are enriched due to RON activation compared to sfRON expression. **C.** Cancer specific genes which are up and down regulated in each cell line

After demonstrating that expression of various RON isoforms expression had differential effects on both cellular oncogenic phenotypes and downstream signaling, we hypothesized that they may have different downstream effectors. The transcriptome wide consequences of RON activation have not been previously reported, and we therefore sought to understand these changes. We utilized RNA sequencing (RNA-seq) to examine the differential gene expression between cell lines that express RON isoforms. We utilized the HPDE cell line where RON is expressed but not phosphorylated and compared it to HPDE cells stimulated with MSP ligand, and those expressing sfRON, RON P5P6, and RONΔ165. We analyzed 3 different RNA specimens from each cell line, and all met quality control standards except for one sample from HPDE stimulated with MSP (Supplementary Figure S6). Heat map of gene specific expression levels show clear pattern differences in genome wide transcription when full length RON is activated (Figure 6A). Interestingly, changes in gene expression induced by exposure to MSP are very similar to those observed following expression of RONΔ165. In contrast, RON P5P6 and sfRON have distinct patterns of gene expression from each other and from HPDE cells stimulated with MSP or expressing RONΔ165. These genome wide patterns in differential expression were then examined using DAVID gene ontology functional annotation which captured all pathways with a false discovery rate (Benjamini) of less than 5% (Figure 6B). Non-coding RNA pathways were upregulated when full length RON alone was activated as were phosphoprotein and ribosome genesis pathways. Short form RON had diverse effects on signaling peptides, secreted proteins, and the extracellular space proteins. RON P5P6 expression upregulated phosphoprotein pathways and downregulated developmental and embryonic signaling pathways (Supplementary Figure S7A).

Examining the list of differentially expressed genes reveals alteration of genes previously associated with cancer (Figure 6C). Each list of genes has some commonalities as all isoforms downregulate DAPK1 and BMP7. Full length RON activation cause increases in ESM1 (endocan) that has previously been associated with pro-angiogenic cytokine production and cancer metastasis [27] and MMP3, which is associated with increased cell tumorigenicity [28]. Short form RON upregulates mir146a, a microRNA with numerous ascribed activities, including regulation of the innate immune system, anti-tumor immune suppression and activation of Notch signaling. It has also been implicated as a tumor suppressor found to work with the BRCA protein to downregulate EGFR [29]. Concordant with that finding, we noted that total EGFR is reduced in the HPDE sfRONe cell line. G-CSF (CSF3) is also overexpressed which can induce activation of myeloid derived suppressor cells which act to create an immunosuppressive environment [30]. Both tumor suppressor genes BMP7 and DAPK1 are downregulated in the absence of MSP in all 3 isoform overexpressing cell lines [31]. This data has been validated using RTPCR to confirm upregulation of mir146a and G-CSF as well as downregulation of BMP-7 in sfRON expressing cells (Supplementary Figure S8). We have previously shown that RON P5P6 isoform is oncogenic, TNFRSF6B (DcR3 protein) prevents TRAIL induced apoptosis [32] and GDF15 (MIC-1 protein) is being investigated as a highly specific marker of pancreatic cancer [33]. GAS1 is a tumor suppressor involved in hedgehog signaling and RUNX3 is a transcription factor that is lost in pancreatic cancer and modulates metastatic potential [34, 35]. Ultimately genome wide RNA sequencing revealed that RON isoform expression produces a pattern of gene expression that differs from the wild type protein.

## DISCUSSION

RON is highly expressed in pancreatic cancer and pre-clinical models suggest its potential as a novel therapeutic target. In order to maximize the potential efficacy of RON directed therapies, a thorough understanding of RON biology is required. As such, we sought to characterize the expression of RON isoforms in human pancreatic cancer and to understand their regulation and distinct functions. We have shown that RON transcript is overexpressed compared to normal pancreas, approximately 40 fold in low RON expressing tumors and as great as 2500 fold in high expressing tumors. As RON is increasingly expressed, there is a concomitant increase in isoform expression with nearly 50% of the transcripts attributed to isoforms. Cancers that express RON (95% in our cohort) express a wide variety of isoforms with a high prevalence of RONΔ165, short form RON, and RON P5P6. This data parallels what the ENCODE project has found; that splice isoforms do not follow a minimalistic pattern and express multiple isoforms simultaneously [9]. We are the first to report quantification of RON transcripts in pancreatic cancer specimens, and the method we used is an averaging of all cells within the tumor tissues. Given that pancreatic cancers have such a profound stromal content and that expression of isoforms likely occurs within only the epithelial malignant cells, our results are almost certainly biased in a negative direction, suggesting that RON isoform expression is likely even higher than what we have detected. It is also possible that specific isoform expression is elevated in a distinct subpopulation of cells that is averaged out when analyzing the tissue as a whole. We would optimally like to quantitate RON protein isoform expression, but it is currently not possible to determine due to similar molecular weights and the lack of specific antibodies to these splice variants.

Our studies suggest that constitutively active isoforms such as sfRON have biologic activity that may have clinical relevance. In a prior study, we demonstrated that P5P6 has transforming activity and in our current study demonstrate that sfRON expression induces oncogenic phenotypes in vitro and is tumorigenic in immortalized pancreatic duct epithelial cells. Similar to previous reports in breast cancer, we found that sfRON expression increases signaling through the AKT pathway [16]. However not all RON isoforms have this effect as RONΔ165 is active but did not markedly affect downstream signaling or enhance invasive abilities. Though sfRON expression induced a clear EMT phenotype in breast cancer cells, we did not observe the same results in pancreatic ductal epithelial cells. *In vitro* we noted only an increase in vimentin and no change in morphology. However, *in vivo* there were areas of low E-cadherin expression and a shift in cell morphology toward a more mesenchymal cell type. SfRON expression may promote epithelial cell interactions with the surrounding stroma and it is possible this is required for phenotypic changes to be occur.

RON directed monoclonal antibodies have shown promise in preclinical studies as they can block MSP induced receptor activation, decrease MAPK and AKT signaling and tumor xenograft growth [7]. We demonstrate that while antibodies such as RON8 effectively inhibit MSP dependent flRON activation, not surprisingly it fails to inhibit constitutive activation. This is almost certainly due to the fact that in sfRON in particular, the extracellular domain is absent, eliminating the antibody target and isoform activation is MSP independent. Comparing RON to EGFR as a prototypical tyrosine kinase in a model [36], receptors are in dynamic state of monomers and dimers. As receptor expression increases, time spent in the dimerization phase increases and receptors are primed for ligand binding. Yet RON isoforms are activated on dimerization, not on ligand binding, likely due to conformational changes. RON 8 does not inhibit dimerization and therefore cannot act to inhibit isoform constitutive activity. This is important clinically as tumors that express high levels of isoforms may escape RON8 treatment effect on full length RON by constitutively active RON isoform signaling. A monoclonal antibody Zt/f2 developed by the Wang group is a specific and effective inhibitor of flRON as well as isoform RONΔ160 but employs a different strategy [37]. It acts by binding to the juxtamembrane region which does not block ligand binding but instead induces RON internalization to decreases cell surface expression and activation. Antibody targeting strategies that inhibit RON dimerization may be more effective on RON isoforms as they will inhibit inappropriate dimerization and activation.

Small molecule RON targeted inhibitors have shown promising pre-clinical efficacy at reducing tumor growth [17, 38]. These compounds are competitive ATP inhibitors that have receptor specificity in the nanomolar range, but hit a wide variety of tyrosine kinases targets at higher concentrations. We tested a new compound LDC047896 for efficacy and found that constitutive kinase activity is blocked at the 100nM to 1 μM range for flRON as well as RON isoforms. Blocking kinase phosphorylation in RON isoforms decreased MAPK signaling but did not affect AKT signaling. It is interesting that ligand binding kinase activity was blocked by LDC047896, but its effect on AKT activation was not. This implies that MSP binding effect on the AKT pathway is kinase domain independent, or that pancreatic cancer cells utilize compensatory mechanisms to activate AKT in the setting of RON kinase inhibition. Viability was significantly decreased solely in RON isoform expressing cell lines demonstrating that the drug effect is specific to RON dependent cell lines. Our findings are consistent with studies of leukemia cell lines expressing sfRON that show that small molecule inhibition reduced viability of sfRON expressing cells only [39].

Epigenetic therapies such as DNA demethylating agents and histone deacetylase inhibitors are currently in clinical trials for treatment of solid tumors [40, 41]. Low doses can reprogram malignant epithelial cells to produce a potent antitumor response and decrease subpopulations of cancer stem cells without significant cytotoxic activity [42]. We chose to study epigenetic therapies because previous studies found that pancreatic cancers which overexpress RON have a hypomethylated promoter compared to the normal pancreas [21]. Decreased methylation of the promoter during carcinogenesis results in RON overexpression and subsequent isoform generation. We treated these cell lines with demethylating agents and found RON expression subsequently decreased in those cell lines that normally overexpressed it. As the promoter for RON is already demethylated, this effect is likely due to effects on transcription factors regulating RON expression and not due to direct effect on the RON promoter itself. In cells which normally do not express RON, and have hypermethylated promoters, we see the expected induction of RON expression with demethylation therapy. Our results are similar to that observed in leukemia cell lines where Azacitidine reduced sfRON but not flRON expression [39]. Reducing RON expression in cell lines which highly express it may have the unintended consequence of reducing sensitivity to RON directed therapies, a hypothesis which bears testing.

To our knowledge, we are the first to report whole transcriptome RNA sequencing when RON or its isoforms is activated. The patterns of gene expression reveal clear differences between flRON and the P5P6 and sfRON isoforms. This finding is further demonstrated by gene ontology analysis that finds modulation of distinctly different pathways. We have presented several specific genes that may contribute to the oncogenic effects of RON or its isoform expression. The RNA-seq data clearly suggests that RON isoforms regulate multiple transcriptional programs related to oncogenesis.

In summary, we have found that biologically active RON isoforms are commonly expressed in pancreatic cancer and that RON is overexpressed at up to 2500 fold higher in pancreatic cancer than normal pancreas with isoform expression comprising nearly half the transcript in some tumors. Future work will focus on understanding the downstream effects of RON isoform signaling which appear to be distinct from the full length protein. Though each contains an active kinase domain, the change in amino acid sequence likely causes changes in secondary conformation to affect how the isoform interacts with other proteins. Small molecules inhibitors block activation of the kinase domain, however the action of RON isoforms may involve unique dimerization partners that may not be inhibited. We have found that traditional inhibition of ligand binding by monoclonal antibodies has no effect on kinase constitutive activation while small molecule inhibitors are effective. DNA demethylation modulates total protein expression and decreases RON isoform expression levels. These results show that RON isoform response to therapeutics warrants consideration when evaluating for efficacy and may serve as biomarkers for predicting response to RON directed therapies.

## MATERIALS AND METHODS

### PCR

Trizol (Invitrogen) was used to extract total RNA from PDXs and Cell lines. A cDNA library was created using SuperScript III Reverse Transcriptase (Invitrogen). End point PCR was the performed using RedTaq DNA Polymerase (Sigma) and resolved using 2% agarose gel electrophoresis. The primers used are listed in Supplementary Table S1.

### Quantitative real-time PCR

All reactions were performed using SsoFast EvaGreen Supermix (Bio-Rad), primers (375nM final concentration), 1 μL of cDNA (corresponding to 73 ng of RNA). Absolute quantification was performed using methods previously described [43]. A plasmid containing the full length RON, RON P5P6, and RONΔ165 cDNA was used to generate a standard curve using specific primers. The equation DNA MW (g) = ((# of bp) × (660 g/mol/bp))/6.022 × 10^23^ particles/mol was used to convert plasmid size to weight. Weight was used to construct a serial dilution of 10^7^ − 10^2^ plasmid copies. Standards were run in duplicate on each plate used to analyze unknown samples and linear standard curve was generated (Supplementary Figure S2). The RON C-terminal standard curve was applied to sfRON unknowns as there are no specific sequences for standardization. The slope of the lines corresponds to the efficiency of the reaction using equation Efficiency = (10^−1/slope^−1) × 100%.

### Human tissues and patient derived xenografts (PDX)

All human tissues were collected and utilized in accordance with IRB approved and IACUC approved protocols at the University of California, San Diego. Human pancreatic cancer tissue was implanted fresh after resection into the pancreas of NOD SCID Gamma (NSG) mice as described previously [44, 45]. PDX's were established and passaged after they were at least 10mm in size. Low passage (less than 5) tumors were used for all analyses.

### Cell culture

The Human Pancreatic Ductal Epithelial (HPDE) cell line was a generous gift from Dr. Ming Tsao and the University Health Network [25, 46]. The HPDE cell line was cultured in Keratinocyte-SFM (Gibco) media with supplements. The MiaPaca, BxPc3, AsPC1, and FG cell lines were obtained from ATCC and cultured in DMEM or RPMI supplemented with 10% FBS. The 34E and 79E cells lines are epithelial cells that were explanted from human pancreatic cancer xenografts. Live cell fluorescent imaging and fluorescent immunohistochemistry imaging was performed using the Nikon A1R Confocal TIRF STORM Microscope and NIS-Elements imaging software. Cells were cultured in 1 well chamber slides (Lab-Tek) after a coating of Poly-D-Lysine was applied (Sigma). RON8 was generously provided by Eli Lilly. LDC047896 was generously provided by Lead Discovery Center GmbH. For RON8 treatment cells were plated in 10mm dishes and grown to 80% confluency. RON8 at 100 ng/mL was used for the defined time periods. Before MSP stimulation (100 ng/mL) HPDE cells were cultured for 24 hours with RON8 (100 nM) and then stimulated for 30 minutes. Treatment with Decitabine lasted for 96 hours with media and drug was changed every 48 hours.

### Molecular cloning and lentiviral transfection

The sfRON-eGFP and RONΔ165-eGFP protein transcript was created by recreating the splicing event using the full length RON cDNA which had previously been attached to GFP. The sfRON construct was created by removing the cDNA region upstream of exon 11. For RONΔ165 the region between exons 10 and 18 was amplified from a primary pancreatic cancer sample using primers listed in Table 1. The PCR amplicon was TA cloned using a PCR Cloning Kit (Qiagen) and competent DH5α E. Coli (Invitrogen). The full length RON+eGFP vector and the RONΔ165 containing vector were cut with specific endonucleases and ligated using T4 DNA Ligase (NEB). DNA sequencing confirmed the correct RONΔ165 sequence and the cDNA was then transferred into a lentiviral plasmid for use in transfection. Isoform cDNA plasmids were packaged using Lipofectamine 2000 (Invitrogen) per protocol. After transfection selective puromycin media was used and the TE300 inverted fluorescence microscope (Nikon) was used to screen for green fluorescence.

### Immunoprecipitation, western blot, and immunohistochemistry

Human pancreatic cancer specimens of approximately 100mg or cultured cells at approximately 80 % confluency were used to make protein lysates in RIPA buffer. Immunoprecipitation (IP) was performed using RON β C-terminal (C20, Santa Cruz). For western blot primary antibodies used are RON β, Phosphotyrosine (4G10, Millipore), Actin (A2066, Sigma), Phospho-AKT (9271, Cell Signaling), AKT (9272, Cell Signaling), Phospho- p44/42 MAPK (9101, Cell Signaling), p44/42 MAPK (9102, Cell Signaling), Vimentin (550513, BD Pharmingen), alpha smooth muscle actin (ab5694, Abcam), E-cadherin (610182, BD), and DNMT1 (C17, Santa Cruz).

### Functional assays

Proliferation assays was performed in triplicate using Thiazolyl Blue Tetrazolium Bromide (1 mg/ml MTT, Sigma. For scratch wound assays, 1×10^6^ cells were plated in a 6 well dish and allowed to attach and grow to confluency. A 1 mL aspirating pipette tip was used to create 3 vertical scratches in each well. Images were taken at 4x magnification with Nikon TE300 inverted fluorescence microscope. Metamorph (Molecular Devices) analysis software was used to outline and determine wound area. Scratch assay was performed three separate times and all data combined. For VEGF assay, the Quantikine human VEGF ELISA (R&D systems) kit was used per protocol. Genomic DNA methylation was assessed using the MethylFlash Methylated DNA Quantification Kit (Epigentek) per manufacturer's specifications

### Orthotopic injections

1×10^6^ HPDE and HPDE sfRONe cells were re-suspended in 10 μL of PBS and mixed with 10 μL of Matrigel (BD). NSG mice were injected orthotopically using a 30 gauge needle. Injections were performed using same protocol described in the orthotopic xenografting section. Mice were monitored for signs of distress and disability throughout the entire experiment. After 13 weeks mice were sacrificed and tumors were harvested. Tumor volume was measure using the equation: Volume = 1/2(length × width^2^). Mice and tumors were imaged using MVX10 (Olympus) camera and cellSens (Olympus) software.

### RNA-sequencing

Three separate plates of the same cell line were grown to 70% confluency and whole RNA was extracted. For MSP stimulation, 100 ng/mL was placed in the media and allowed to incubate for 12 hours before RNA lysates were made. Total RNA was assessed for quality using an Agilent Tapestation, and samples with an RNA Integrity Number (RIN) greater than 8 were used to generate RNA libraries using Illumina's TruSeq V2 Sample Prep Kit. RNA libraries were multiplexed and sequenced with 50 basepair (bp) single end reads (SR50) to a depth of approximately 36 million reads per sample on an Illumina HiSeq2000. The transcriptome of each sample was analyzed using RPKM method to generate counts for each Ensembl gene ID. Differential gene expression between sample groups was calculated using DESeq method. We then filtered the analysis by those genes whose counts were greater than 0, False Discovery rate < 0.05 significance, and fold change less than −3 or greater than 3. This generated a gene list of approximately 1000 genes per sets of comparisons. Expression levels were then plotted using heatmap compared to inactive RON. Gene lists were created as up or downregulated and the DAVID gene ontology functional annotation [47]was used with a Benjamini false discovery rate of 5% (5E-2) as the maximum.

### In vitro kinase assays

RON kinase assay was carried out as a 384-well FlashPlate assay with streptavidine coated plates Perkin Elmer (Waltham, MA). 4.5nM GST tagged human recombinant RON kinase (Life Technologies; Carlsbad, CA), 500 nM biotinylated peptide substrate RDILDREYYSVQQHRH-amide (autophosphorylation site derived peptide substrate, custom-made) and 2 μM ATP (with 0.5 pCi 33P-ATP/well) were incubated in a total volume of 50 μl reaction buffer (50 mM HEPES, 5 mM MgCI2, 2 mM DTT, 0.1% BSA, 0.01 % Igepal CA630, 1% DMSO, pH 7.5) in the presence or absence of test substance at 22°C for 30 min. Radioactivity was measured using a Topcount scintillation counter (PerkinElmer; Waltham, MA). The IC50 values were calculated using the software AssayExplorer (Elsevier; Waltham, MA).

Biochemical cMet inhibition was measured in a 384 well flash-plate assay format as previously described [48]. Briefly, His6-tagged recombinant human cMet kinase domain (aa 974–end; 20 ng) and biotinylated poly-Ala-Glu-Lys-Tyr (6:2:5:1; 500 ng) were incubated with or without the test compound for 90 minutes at room temperature in 100 μL buffer containing 0.3 μCi 33P-ATP, 2.5 μg polyethylene glycol 20.000, and 1% dimethyl sulfoxide (DMSO). Radioactivity was measured with a TopCount microplate scintillation and luminescence counter (Packard BioScience BV). Inhibitory 50% concentration values (IC50) were calculated by nonlinear regression analysis using the RS/1 software program.

### Cellular kinase assays

The murine fibroblast cell line NIH-3T3 was engineered to stably express the TPR-RON fusion protein consisting of the N-terminal part of TPR and the C-terminal kinase domain of RON [49]. Ligand independent constitutive phosphorylation of the RON kinase domain was confirmed by Western blot using a phospho specific antibody detecting RON when phosphorylated at Y1238/Y1239 (AF1947; R&D Systems; Minneapolis, MN). Cellular RON potency of inhibitors was assessed by a phospho-TPR-RON luminex assay. Levels of phospho-TPR-RON Y1238/Y1239 were quantified by incubation of lysates with microspheres coupled to total TPR antibodies (#WH0007175M1, Sigma-Aldrich; St. Louis, MO) overnight. Amounts of phospho-TPR-RON Y1238/Y1239 were measured on a Luminex 200 machine (Luminex; Austin, TX) according to the manufacturer's instructions. Level of phospho-TPR-RON Y1238/Y1239 were calculated as percent of DMSO controls, and IC50 values were determined using non-linear regression with the software Graph Pad Prism (Statcon; Witzenhausen, Germany).

Total cMet phosphorylation was assessed by cMet capture ELISA in Nunc-Immuno MicroWell 96-well solid plates (Sigma-Aldrich; St. Louis, MO) as previously described [48]. Briefly, A549 human lung cancer cells were seeded 2 days before treatment, serum-starved for 20 hours, and treated on day 3 with tset substances or DMSO control for 45 minutes at 37°C, 5% CO2. Upon stimulation with 100 ng/mL HGF for 5 minutes, cells were lysed with 70 μL per well ice-cold lysis buffer (20 nmol/L HEPES, pH 7,4; 10% (V/V) Glycerol; 150 nmol/L NaCl; 1% (V/V) Triton-X-100; 2 nmol/L EDTA) supplemented with protease and phosphatase inhibitors. In the ELISA, the capture antibody was specific for the cMet extracellular domain, whereas an antiphosphotyrosine biotin-labeled antibody was used for detection. Tyrosine phosphorylation was revealed using a streptavidin peroxidase conjugate and chemiluminescence read-out

## SUPPLEMENTARY FIGURES AND TABLES




