# Supplementary material for: Characterization of RON protein isoforms in pancreatic cancer: implications for biology and therapeutics
Source: Oncotarget. 2016 Jun 14;7(29):45959–75. doi: 10.18632/oncotarget.10009 (PMC5216774; doi:10.18632/oncotarget.10009)
Supplement: Supplementary file 1 [file oncotarget-07-45959-s001.pdf]

Characterization of RON protein isoforms in pancreatic cancer: implications for biology and therapeutics

SUPPLEMENTARY FIGURES AND TABLES

Sequence 500-4F

GACATTTAGGTGACTATAGAATACAGCGGCCGCGAGCTCGGGCCCCACACGTGTGGTCTAGAGCTAGCCTA
GGCTCGAGAAGCTTGTCGACGAATTCAGATT**GTGGGTATCAACGTGACCGT**GGGTGGTGAGAGCTGCCAGC
ACGAGTTCCGGGGGGACATGGTTGTCTGCCCCCTGCCCCCATCCCTGCAGCTTGGCCAGGATGGTGCCCCA
TTGCAGGTCTGCGTAGATGGTGAATGTCATATCCTGGGTAGAGTGGTGCGGCCAGGGCCAGATGGGGTCCC
ACAGAGCACGCTCCTTGGTATCCTGCTGCCTTTGCTGCTGCTTGTGGCTGCACTGGCGACTGCACTGGTCT
TCAGCTACTGGTGGCGGAGGAAGCAGCTAGTTCTTCCTCCCAACCTGAATGACCTGGCATCCCTGGACCAG
ACTGCTGGAGCCACACCCCTGCCTATTCTGTACTCGGGCTCTGACTACAGAAGTGGCCTTGGTGAGATAGT
GGAGGCATGAGAAGCTAAAGCCACCCCCTGTCCCTACAAGCTCCTCATTCCCTCTCCCCACAG**CACTCCCT**
GCCATTGATGGTAATCACGAATTCTGGATCCGATACGTAACGCGTCTGCAGCATGCGTGGTACCGAGCTTT
CCCTATAGTGAGTCGTATTAGAGCTTGGCGTAATCATGGTCATAGCTGTTTCCTGTGTGAAATTGTTATCC
GCTCACAATTCCACACAACATACGAGCCGGAAGCATAAAGTGTAAAGCCTGGGGTGCCTAATGAGTGAGCT
AACTCACATTAATTGCGTTGCGCTCACTGCCCGCTTTCCAGTCGGGAAACCTGTCGTGCCAGCTGCATTAA
TGAATCGGCCAACGCGCGGGGAGAGGCGGTTTGCGTATTGGGCGCTCTTCCGCTTCCTCGCTCACTGACTC
GCTGCGCTCGGTCGTTCGGCTGCGGCGAGCGGTATCAGCTCACTCAAAGGCGGTAATACGGTTATCCACAG
AATCAAGGGATAACCGAGGAAAAGAACATGTGAGCAAAAGGCCAACAAAAGGCCAGGAAACCGTAAAAAAG
GCCGCGTTGCTGGCGTTTTTTCCATAGGCTTCCCCCCCCTTGACGAGCATCAACAAAAAATCACCCCTCAA
GTTCAAAAGTTGGCGAAAACCCAACGGGAATTATAAAAAATACCAGGGCTTTTTCCCCCTTGGAAAGTTCC
CTTCGTGGCCCCTCTCCTTTTTCCCCAACCCTTCCCCCCTTACCCCGAAACCCTTTTTCCCCCCTTTTCTC
CCCTTTCCGGAAAAGGGGGGGCCCCTTTTTTTCTAAAGCCCTCACCCTTTTTAGGGTTT

Bold – Primers
Yellow Highlight – T wild type, mutated to C in other sequencing samples
Green Highlight – intron 13 insertion

Supplementary Figure S1: Sequencing of intron 13 insertion from PCR products.

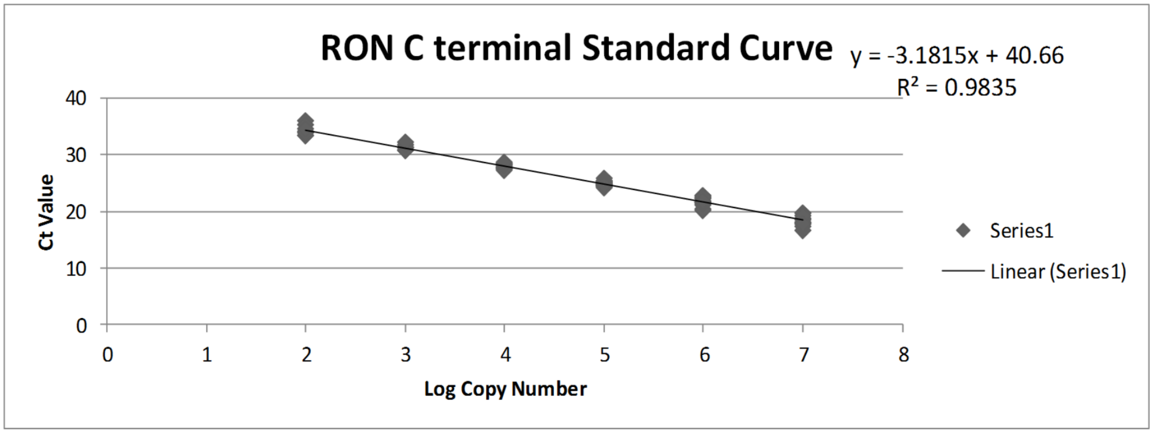

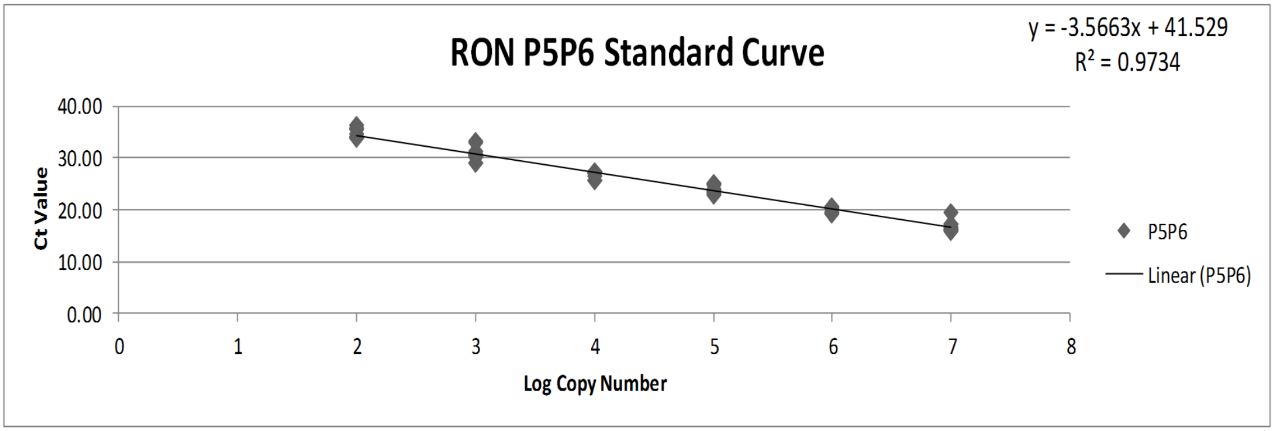

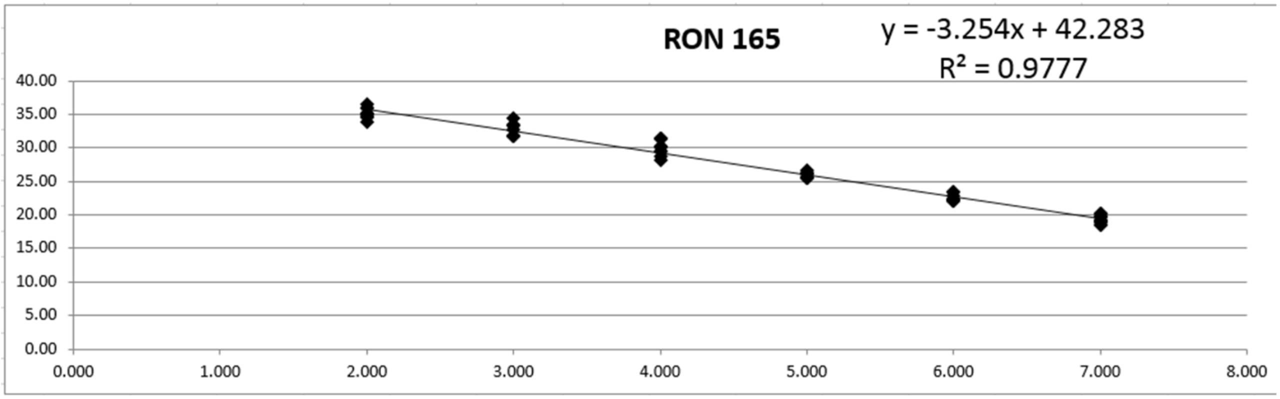

Supplementary Figure S2: Primer specific standard curves used for absolute quantitative PCR.

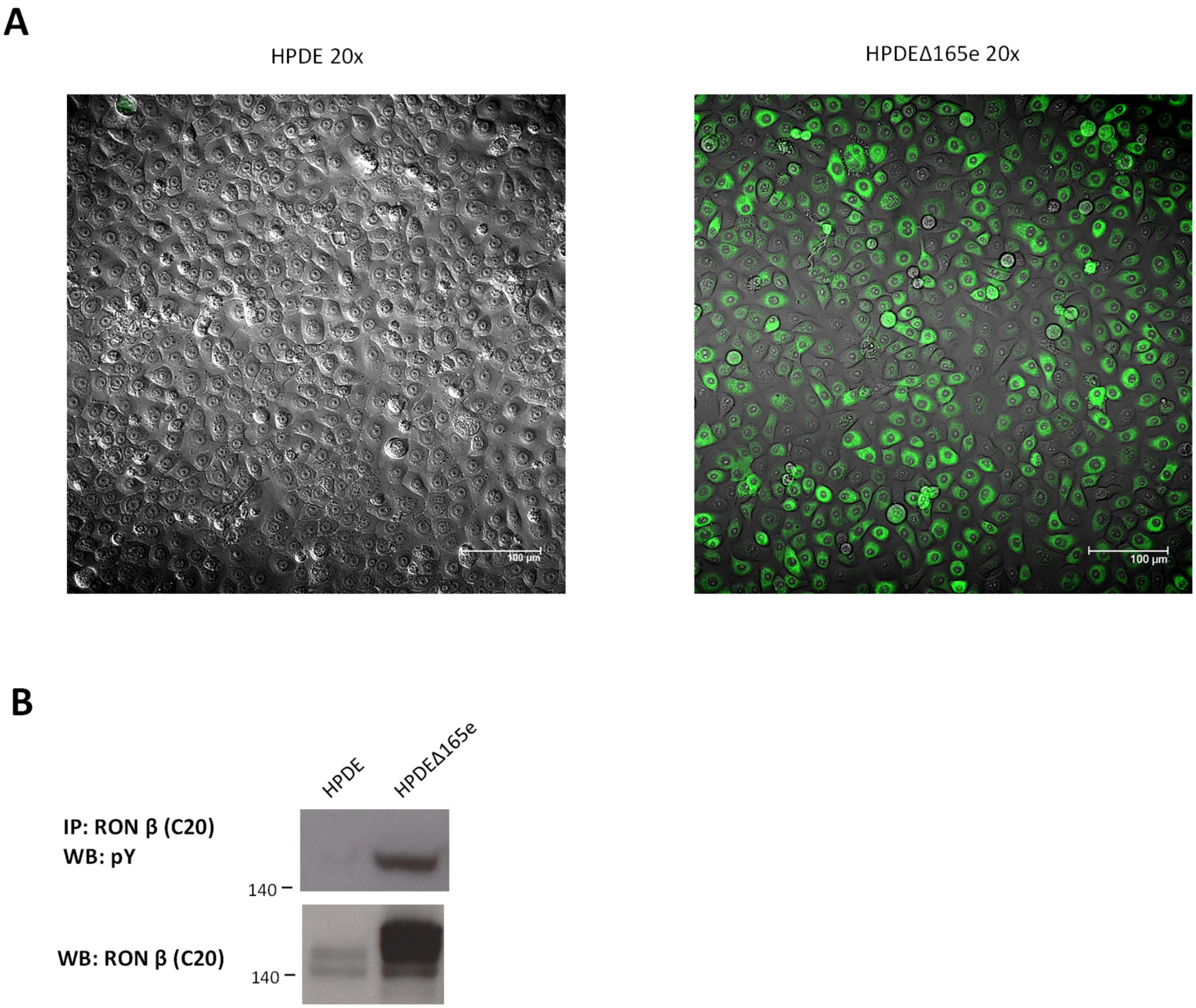

Supplementary Figure S3: A. RONΔ165 does not alter cell morphology when expressed in HPDE cells. **B.** HPDEΔ165 is constitutively active.

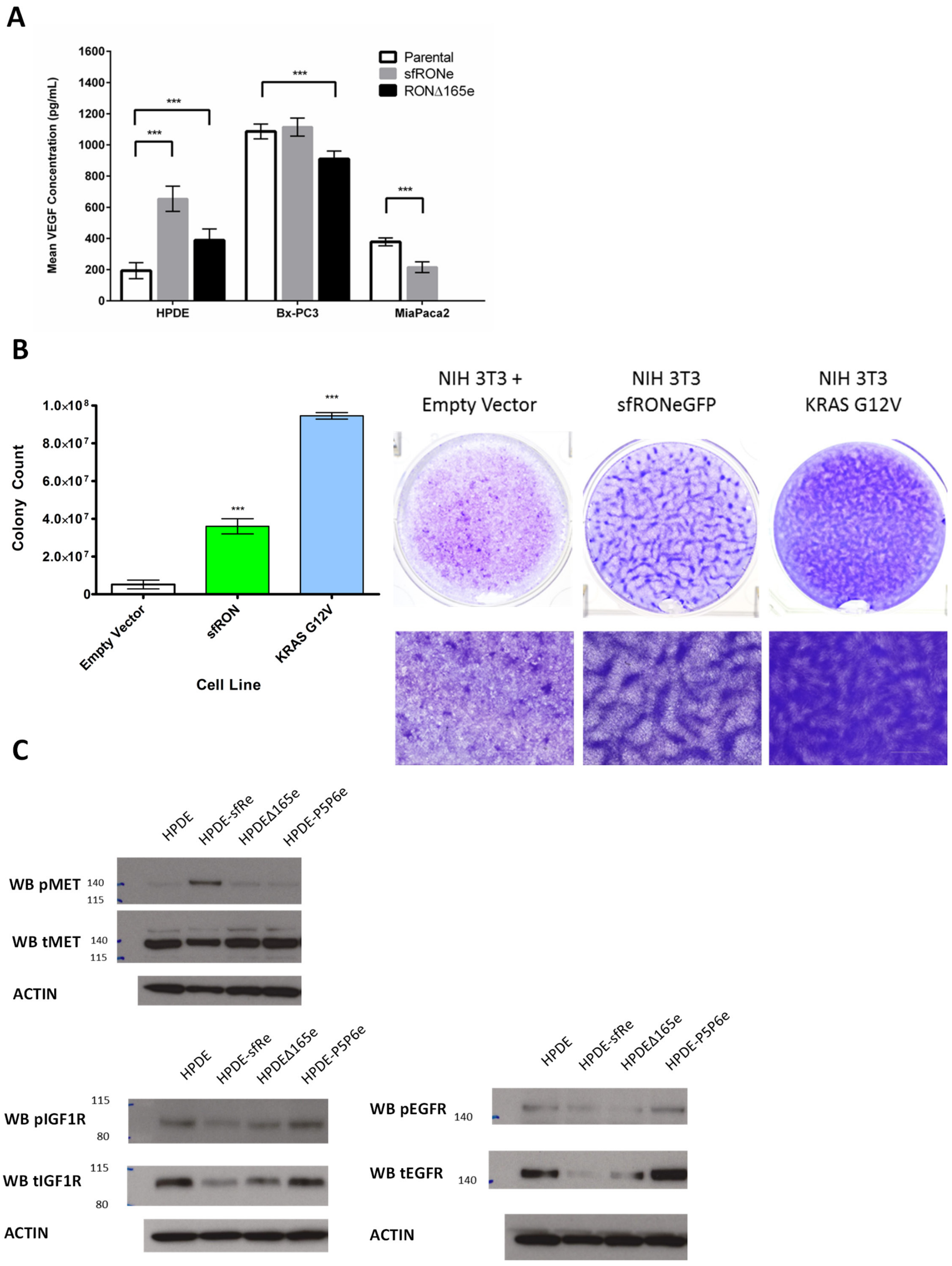

Supplementary Figure S4: A. VEGF secretion as a result of sfRON and RONΔ165 expression in 3 separate pancreatic cell lines. **B.** Cell transformation assay using NIH 3T3 cells shows increased colony formation with sfRON expression however not as much as KRAS G12V expression. **C.** RON isoform expression induces phosphorylation of MET but not IGF1R and EGFR.

A

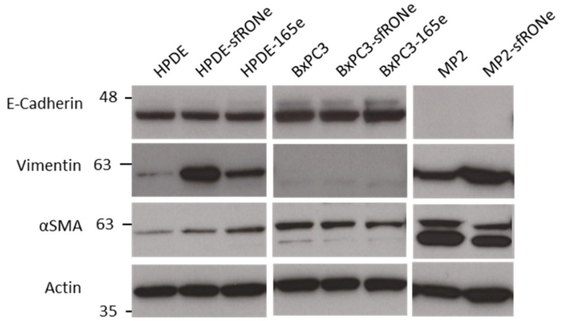

B

RONΔ165 (HPDE-165e) vs inactive RON (HPDE)	Gene Expression Fold Change	Raw p-value	False Discovery Rate
CDH1 (e-cadherin)	-1.3809	0.0055	0.0244
CDH2 (n-cadherin)	3.2848	3.00E-06	3.14E-05
ACTA2 (alpha SMA)	-2.2885	1.68E-10	4.24E-09
VIM (Vimentin)	1.8141	3.46E-07	4.43E-06
SNAI1(Snail)	1.3664	0.1397	0.3637
SNAI2(Slug)	-1.0188	0.8764	1
ZEB1	7.4332	0.0008	0.0043
TWIST1	1.1437	0.4348	0.822
KHDRBS1 (Sam68)	1.5346	0.0002	0.0015

C

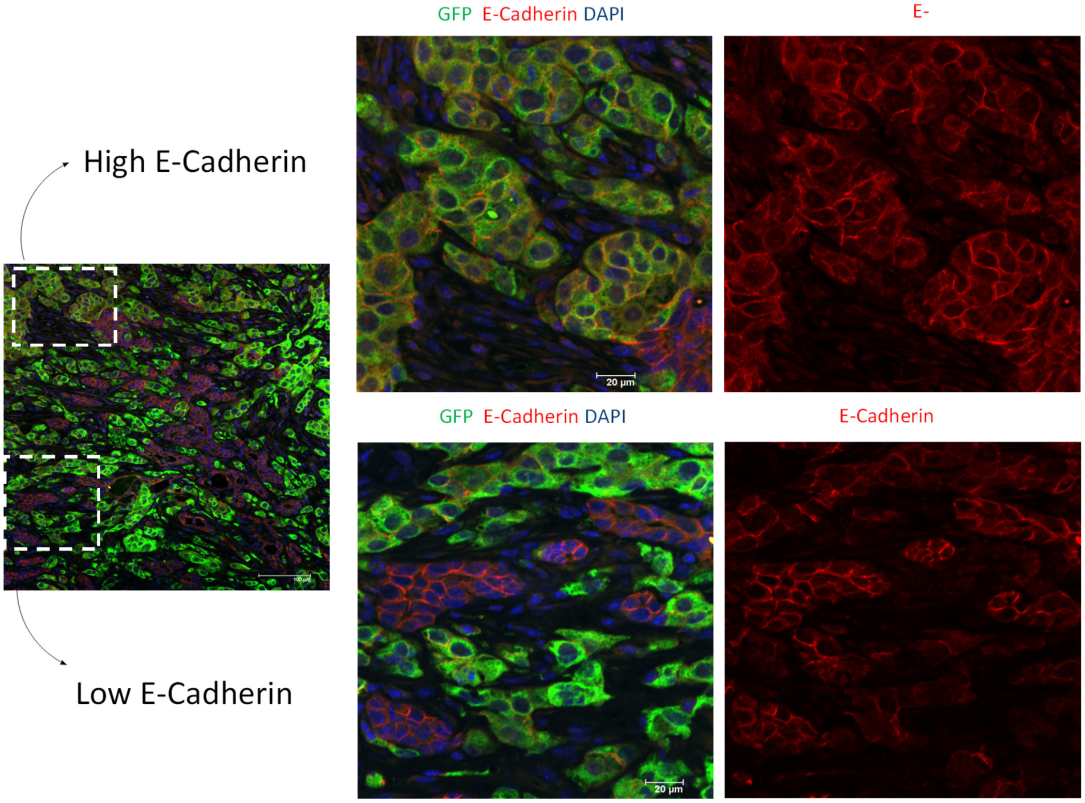

Supplementary Figure S5: A. Short form RON has been implicated in epithelial to mesenchymal transition in other cancer types but did not induce a switch in pancreatic cancer cell lines. **B.** Differential gene expression using RNA-seq comparing HPDEΔ165 to HPDE cell lines showed no evidence of epithelial to mesenchymal transition **C.** Tumors showed differential expression of E-cadherin and cell morphology. Short form RON expressing cells in the tumor had areas of high E-Cadherin and round morphology (Top panels) and areas of low E-Cadherin and more mesenchymal morphology (bottom panels, arrow).

A

ID	Condition	Treatment
Sample_HDPE_1	Inactive RON	Control
Sample_HPDE_2	Inactive RON	Control
Sample_HPDE_3	Inactive RON	Control
Sample_HPDE+MSP_1	Active full length RON	MSP stimulation
Sample_HPDE+MSP_2	Active full length RON	MSP stimulation
Sample_HPDE+MSP_3	Active full length RON	MSP stimulation
Sample_HPDE-165_1	Active 165 RON isoform	165 overexpression
Sample_HPDE-165_2	Active 165 RON isoform	165 overexpression
Sample_HPDE-165_3	Active 165 RON isoform	165 overexpression
Sample_HPDE-P5P6_1	Active P5P6 RON isoform	P5P6 overexpression
Sample_HPDE-P5P6_2	Active P5P6 RON isoform	P5P6 overexpression
Sample_HPDE-P5P6_3	Active P5P6 RON isoform	P5P6 overexpression
Sample_HPDE-sfRON1	Active sfRON isoform	sfRON overexpression
Sample_HPDE-sfRON2	Active sfRON isoform	sfRON overexpression
Sample_HPDE-sfRON3	Active sfRON isoform	sfRON overexpression

← Removed from analysis due to not passing QC (Sample_HPDE+MSP_3)

B

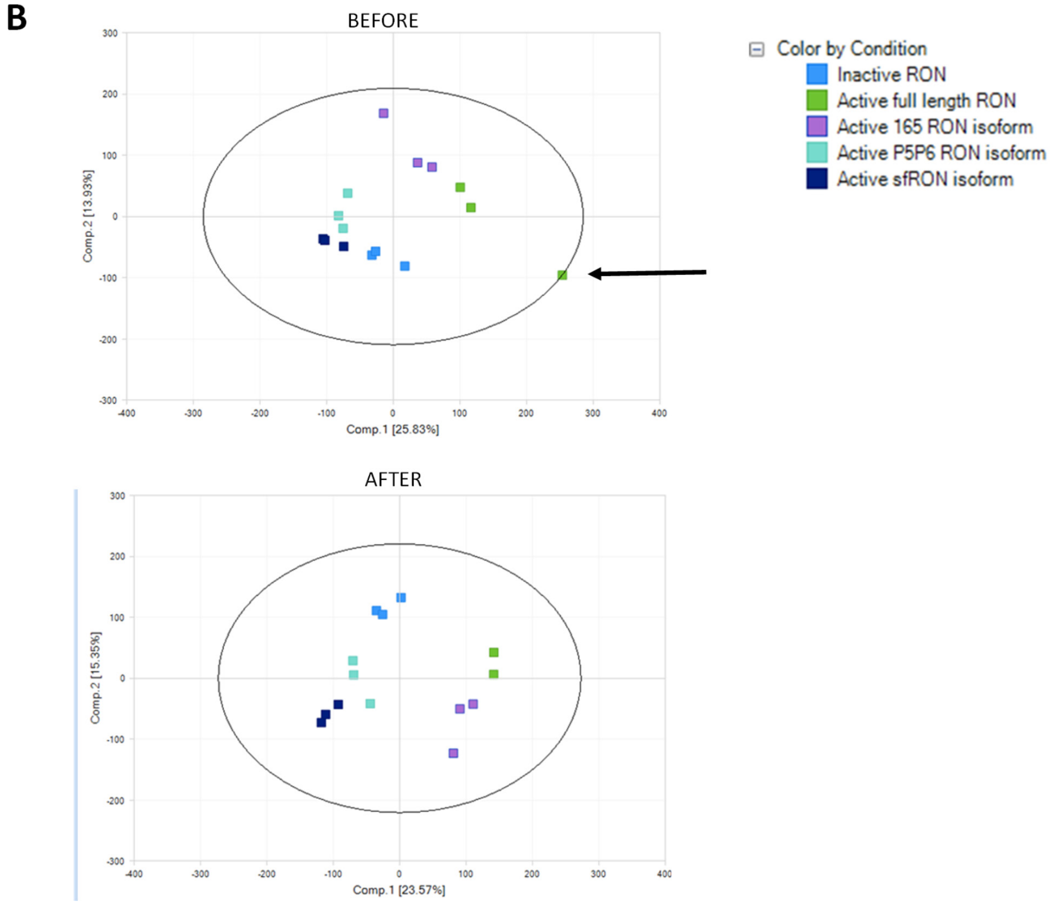

Supplementary Figure S6: A. One sample out of 3 HPDE cells stimulated with MSP was removed from the data analysis. **B.** This sample did not pass quality control and was an outlier on principal control analysis.

A Activated RON Pathways Upregulated

Category	Term	Count	%	P-Value	Benjamini
GOTERM_BP_FAT	ncRNA processing	22	5.7	6.4E-11	1.0E-7
SP_PIR_KEYWORDS	ribosome biogenesis	12	3.1	8.4E-10	3.1E-7
SP_PIR_KEYWORDS	phosphoprotein	188	48.7	3.4E-9	6.1E-7
GOTERM_BP_FAT	ribonucleoprotein complex biogenesis	19	4.9	1.0E-8	8.2E-6
GOTERM_BP_FAT	ribosome biogenesis	16	4.1	1.1E-8	5.9E-6
GOTERM_BP_FAT	ncRNA metabolic process	20	5.2	8.6E-8	3.6E-5
GOTERM_CC_FAT	nucleolus	37	9.6	1.1E-7	2.9E-5
GOTERM_CC_FAT	nuclear lumen	57	14.8	4.9E-7	6.6E-5
GOTERM_BP_FAT	RNA processing	30	7.8	6.2E-7	2.0E-4
GOTERM_CC_FAT	intracellular non-membrane-bounded organelle	84	21.8	1.4E-6	1.3E-4
GOTERM_CC_FAT	non-membrane-bounded organelle	84	21.8	1.4E-6	1.3E-4
INTERPRO	Tubulin/FtsZ, GTPase domain	7	1.8	4.5E-6	2.9E-3
GOTERM_BP_FAT	rRNA processing	11	2.8	9.8E-6	2.7E-3
GOTERM_BP_FAT	rRNA metabolic process	11	2.8	1.4E-5	3.4E-3
SP_PIR_KEYWORDS	rrna processing	9	2.3	1.5E-5	1.8E-3
GOTERM_CC_FAT	intracellular organelle lumen	60	15.5	2.9E-5	2.0E-3
SP_PIR_KEYWORDS	trna processing	9	2.3	3.0E-5	2.8E-3
GOTERM_BP_FAT	protein polymerization	8	2.1	3.7E-5	7.5E-3
INTERPRO	Tubulin/FtsZ, 2-layer sandwich domain	6	1.6	4.8E-5	1.5E-2
GOTERM_CC_FAT	organelle lumen	60	15.5	5.7E-5	3.1E-3
INTERPRO	Tubulin, conserved site	6	1.6	6.0E-5	1.3E-2
PIR_SUPERFAMILY	PIRSF002306:tubulin	6	1.6	6.2E-5	1.0E-2
INTERPRO	Tubulin	6	1.6	7.5E-5	1.2E-2
GOTERM_BP_FAT	tRNA processing	9	2.3	9.7E-5	1.8E-2
GOTERM_CC_FAT	membrane-enclosed lumen	60	15.5	1.0E-4	4.5E-3
GOTERM_CC_FAT	nucleolar part	6	1.6	1.6E-4	6.3E-3
GOTERM_CC_FAT	small nucleolar ribonucleoprotein complex	5	1.3	5.6E-4	1.9E-2

(*Continued*)

sfRON Expression Pathways Upregulated

Category	Term	Count	%	P-Value	Benjamini
SP_PIR_KEYWORDS	signal	125	31.7	2.9E-14	1.2E-11
UP_SEQ_FEATURE	signal peptide	125	31.7	4.7E-14	6.3E-11
GOTERM_CC_FAT	extracellular region part	56	14.2	1.7E-12	4.7E-10
GOTERM_CC_FAT	extracellular space	44	11.2	3.9E-11	5.3E-9
GOTERM_CC_FAT	extracellular region	84	21.3	6.0E-11	5.5E-9
SP_PIR_KEYWORDS	Secreted	74	18.8	1.2E-10	2.3E-8
SP_PIR_KEYWORDS	developmental protein	45	11.4	3.7E-10	4.9E-8
SP_PIR_KEYWORDS	cytokine	20	5.1	3.5E-9	3.4E-7
SP_PIR_KEYWORDS	glycoprotein	134	34.0	1.2E-8	9.7E-7
GOTERM_MF_FAT	cytokine activity	19	4.8	1.0E-7	5.2E-5
UP_SEQ_FEATURE	glycosylation site:N-linked (GlcNAc...)	126	32.0	1.8E-7	1.2E-4
GOTERM_BP_FAT	locomotory behavior	21	5.3	8.7E-7	1.5E-3
GOTERM_BP_FAT	behavior	28	7.1	1.2E-6	1.1E-3
UP_SEQ_FEATURE	DNA-binding region:Homeobox	17	4.3	1.4E-6	6.1E-4
GOTERM_BP_FAT	immune response	34	8.6	4.5E-6	2.7E-3
GOTERM_BP_FAT	taxis	15	3.8	5.0E-6	2.2E-3
GOTERM_BP_FAT	chemotaxis	15	3.8	5.0E-6	2.2E-3
INTERPRO	Homeobox, conserved site	18	4.6	5.9E-6	3.9E-3
SP_PIR_KEYWORDS	Homeobox	18	4.6	6.9E-6	4.5E-4
INTERPRO	Homeobox	18	4.6	7.0E-6	2.3E-3
GOTERM_BP_FAT	defense response	31	7.9	8.7E-6	3.1E-3
SP_PIR_KEYWORDS	disulfide bond	90	22.8	1.4E-5	7.7E-4
SP_PIR_KEYWORDS	pharmaceutical	8	2.0	2.3E-5	1.1E-3
INTERPRO	Homeodomain-related	17	4.3	3.2E-5	7.0E-3
UP_SEQ_FEATURE	disulfide bond	86	21.8	4.1E-5	1.4E-2
SP_PIR_KEYWORDS	angiogenesis	9	2.3	4.6E-5	2.0E-3
SP_PIR_KEYWORDS	chemotaxis	9	2.3	8.6E-5	3.4E-3
SMART	HOX	18	4.6	1.0E-4	1.7E-2
INTERPRO	Small chemokine, C-X-C	5	1.3	1.1E-4	1.7E-2
KEGG_PATHWAY	Cytokine-cytokine receptor interaction	17	4.3	1.1E-4	1.3E-2
GOTERM_BP_FAT	inflammatory response	19	4.8	1.2E-4	3.6E-2
INTERPRO	Small chemokine, C-X-C/Interleukin 8	5	1.3	1.5E-4	1.9E-2
GOTERM_BP_FAT	positive regulation of T cell activation	9	2.3	1.5E-4	3.8E-2
GOTERM_BP_FAT	regulation of locomotion	14	3.6	1.6E-4	3.4E-2
INTERPRO	Small chemokine, interleukin-8-like	7	1.8	1.7E-4	1.8E-2
PIR_SUPERFAMILY	PIRSF002522:CXC chemokine	5	1.3	2.0E-4	3.6E-2
GOTERM_CC_FAT	proteinaceous extracellular matrix	18	4.6	3.3E-4	2.2E-2
SP_PIR_KEYWORDS	extracellular matrix	15	3.8	3.3E-4	1.2E-2

(*Continued*)

sfRON Expression Pathways Downregulated

Category	Term	Count	%	P-Value	Benjamini
SP_PIR_KEYWORDS	Secreted	61	23.5	6.6E-13	2.2E-10
GOTERM_CC_1	extracellular region	64	24.6	2.5E-10	2.7E-9
SP_PIR_KEYWORDS	signal	83	31.9	9.6E-10	1.6E-7
UP_SEQ_FEATURE	signal peptide	83	31.9	1.3E-9	1.2E-6
GOTERM_CC_1	extracellular region part	36	13.8	1.6E-7	8.9E-7
GOTERM_BP_ALL	epidermis development	15	5.8	2.0E-7	2.8E-4
GOTERM_BP_ALL	ectoderm development	15	5.8	5.3E-7	3.6E-4
GOTERM_BP_ALL	tissue development	25	9.6	1.1E-5	5.1E-3
SP_PIR_KEYWORDS	glycoprotein	87	33.5	1.6E-5	1.7E-3
UP_SEQ_FEATURE	short sequence motif:Cell attachment site	9	3.5	2.2E-5	9.5E-3
GOTERM_MF_ALL	calcium ion binding	29	11.2	5.1E-5	2.1E-2
GOTERM_BP_ALL	organ development	44	16.9	5.4E-5	1.8E-2
GOTERM_BP_ALL	system development	54	20.8	5.8E-5	1.6E-2
SP_PIR_KEYWORDS	calcium	26	10.0	6.2E-5	5.1E-3
UP_SEQ_FEATURE	glycosylation site:N-linked (GlcNAc...)	82	31.5	7.1E-5	2.1E-2
SP_PIR_KEYWORDS	extracellular matrix	13	5.0	8.8E-5	5.7E-3

RONΔ165 Expression

446 Genes Pathways Upregulated

Category	Term	Count	%	P-Value	Benjamini
UP_SEQ_FEATURE	region of interest:Beta	5	1.1	4.1E-5	5.3E-2
UP_SEQ_FEATURE	region of interest:Alpha	5	1.1	4.1E-5	5.3E-2
SP_PIR_KEYWORDS	phosphoprotein	197	44.3	4.3E-5	1.6E-2
UP_SEQ_FEATURE	metal ion-binding site:Divalent metal cation; cluster A	5	1.1	6.3E-5	4.1E-2
UP_SEQ_FEATURE	metal ion-binding site:Divalent metal cation; cluster B	5	1.1	6.3E-5	4.1E-2
INTERPRO	Metallothionein, vertebrate, metal binding site	5	1.1	6.6E-5	4.5E-2
PIR_SUPERFAMILY	PIRSF002564:metallothionein	5	1.1	6.7E-5	1.2E-2
SP_PIR_KEYWORDS	metal-thiolate cluster	5	1.1	9.2E-5	1.7E-2
INTERPRO	Metallothionein, vertebrate	5	1.1	9.8E-5	3.3E-2
INTERPRO	Metallothionein superfamily, eukaryotic	5	1.1	9.8E-5	3.3E-2
INTERPRO	WD40 repeat 2	16	3.6	1.4E-4	3.2E-2
INTERPRO	WD40 repeat, region	16	3.6	2.5E-4	4.2E-2
INTERPRO	WD40 repeat, subgroup	16	3.6	3.0E-4	4.0E-2

234 Genes Pathways Downregulated

None significant

(*Continued*)

RON P5P6 Expression

564 Genes Pathways Upregulated

Category	Term	Count	%	P-Value	Benjamini
SP_PIR_KEYWORDS	phosphoprotein	253	44.9	1.6E-5	6.2E-3

205 Genes Pathways Downregulated

Term	RT	%	P-Value	Benjamini	Benjamini
SP_PIR_KEYWORDS	developmental protein	25	12.9	2.4E-7	6.0E-5
GOTERM_BP_ALL	regionalization	13	6.7	4.4E-7	5.3E-4
GOTERM_BP_ALL	pattern specification process	14	7.2	1.8E-6	1.1E-3
GOTERM_BP_ALL	skeletal system development	15	7.7	2.4E-6	9.8E-4
GOTERM_BP_ALL	multicellular organismal development	52	26.8	2.5E-6	7.7E-4
SP_PIR_KEYWORDS	Secreted	36	18.6	4.6E-6	5.8E-4
SP_PIR_KEYWORDS	signal	55	28.4	6.3E-6	5.3E-4
UP_SEQ_FEATURE	signal peptide	55	28.4	7.6E-6	5.3E-3
GOTERM_BP_ALL	developmental process	54	27.8	8.0E-6	1.9E-3
GOTERM_MF_ALL	sequence-specific DNA binding	19	9.8	1.0E-5	3.1E-3
GOTERM_CC_ALL	extracellular region	39	20.1	1.9E-5	3.5E-3
GOTERM_BP_ALL	system development	43	22.2	2.2E-5	4.6E-3
PANTHER_BP_ALL	BP00193:Developmental processes	40	20.6	3.0E-5	3.2E-3
GOTERM_BP_ALL	embryonic development	18	9.3	3.2E-5	5.6E-3
GOTERM_BP_ALL	embryonic morphogenesis	13	6.7	4.1E-5	6.3E-3
GOTERM_BP_ALL	anterior/posterior pattern formation	9	4.6	6.2E-5	8.4E-3
GOTERM_MF_ALL	transcription factor activity	23	11.9	6.9E-5	1.1E-2
GOTERM_BP_ALL	anatomical structure development	44	22.7	7.1E-5	8.6E-3
UP_SEQ_FEATURE	DNA-binding region:Homeobox	10	5.2	7.5E-5	2.5E-2
SP_PIR_KEYWORDS	Homeobox	11	5.7	8.9E-5	5.6E-3
PANTHER_BP_ALL	BP00248:Mesoderm development	17	8.8	9.1E-5	4.9E-3
INTERPRO	Homeobox, conserved site	11	5.7	9.2E-5	3.5E-2
INTERPRO	Homeobox	11	5.7	1.0E-4	2.0E-2
GOTERM_CC_ALL	extracellular region part	23	11.9	1.1E-4	9.6E-3
SMART	HOX	11	5.7	2.1E-4	2.0E-2
PANTHER_BP_ALL	BP00201:Skeletal development	8	4.1	2.4E-4	8.6E-3
GOTERM_BP_ALL	embryonic organ development	9	4.6	2.6E-4	2.8E-2
GOTERM_BP_ALL	embryonic organ morphogenesis	8	4.1	3.0E-4	3.0E-2
PANTHER_BP_ALL	BP00245:Segment specification	7	3.6	4.5E-4	1.2E-2
GOTERM_BP_ALL	organ development	32	16.5	4.5E-4	4.1E-2
PANTHER_BP_ALL	BP00199:Neurogenesis	16	8.2	5.7E-4	1.2E-2
PANTHER_BP_ALL	BP00178:Stress response	9	4.6	6.2E-4	1.1E-2

Supplementary Figure S7: A. DAVID gene ontology pathways significantly (FDR 0.05) up and down regulated when full length RON, sfRON, RONΔ165, or RON P5P6 is expressed.

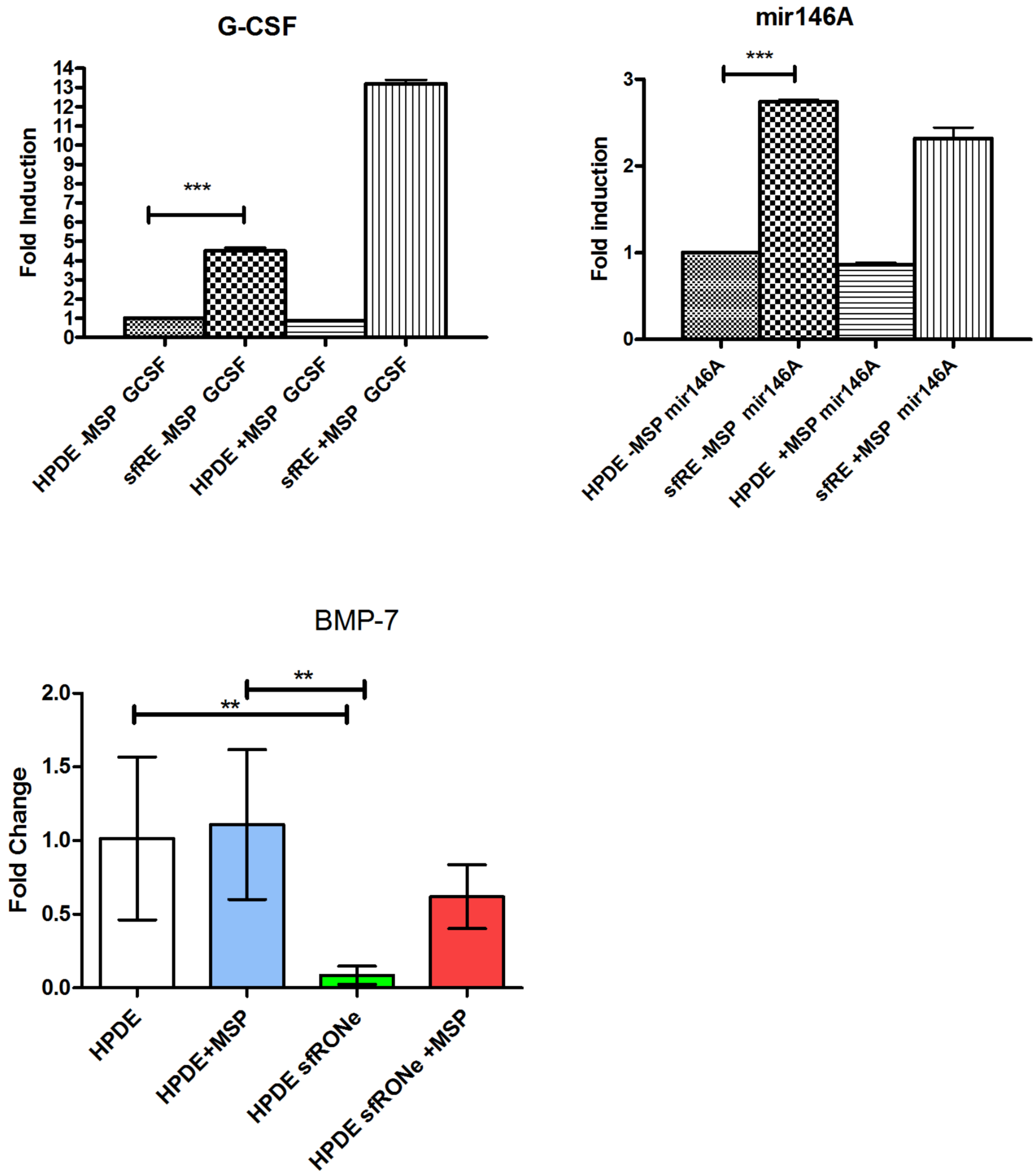

Supplementary Figure S8: RT-PCR of 3 genes which validate that mir146a and G-CSF are upregulated, and BMP-7 downregulated in sfRON expressing cells.

Supplementary Table S1: Primers used for end point and quantitative PCR

Primer	Forward	Reverse
Wild type RON	TAGTGTCTGCACTGCTTGGG	GCTGTTCTGGACGCACATTC
Exon 4-7	GGCATGGCATTTCATGGGCT	AGAGTCTGTCTGTAGGCACC
Exon 10-12	TGCCGCCTTCCTGAATATGT	TCCTTGGTATCCTGCTGCCT
Exon 11 deletion specific (RONΔ165, RONΔ155)	GATGGGTGGCAGGGAATCTG	CCATCTACGCAGACCTCAAACT
Short-form RON specific (RONΔ55)	GACCCTCTCTGCAGTATATTGGG	CCATCTACGCAGACCTGCAA
C-terminal deletion (RONΔ170)	CTGATGTGGTACCAAGTGATGC	TGGCAGCTGCACATAATGGT
Intron 13 Insertion	GAGGAGCATGCCATTAAGTTTGA	GTCCAGCATGCAGTTCCGC
Partial exon 5, Partial exon 6 deletion (P5P6)	GGCAGTACAAGGCCTACCAA	TCAGTCCCATTGACCAGCAC
Actin	AATGTGGCCGACGACTTTGATTGC	AGGATGGCAAGGGACTTCCTGTAA

Supplementary Table S2: Activity of compounds BMS777607 and LDC047896tested at 1 µM across a panel of 240 different kinases. Data are expressed as % effect response versus each kinase tested at its respective Km for ATP; %inhibition = %effect * (-1). Profiling of compounds has been performed at Merck Millipore, Germany.

See Supplementary File 1
