# Supplementary material for: Characterization of RON protein isoforms in pancreatic cancer: implications for biology and therapeutics
Source: Oncotarget. 2016 Jun 14;7(29):45959–75. doi: 10.18632/oncotarget.10009 (PMC5216774; doi:10.18632/oncotarget.10009)
Supplement: Supplementary file 2 [file oncotarget-07-45959-s002.docx]

**Supplemental Table 2:** Activity of compounds BMS777607 and LDC047896 tested at 1 µM across a panel of 240 different kinases. Data are expressed as %effect response versus each kinase tested at its respective Km for ATP; %inhibition = %effect * (-1). Profiling of compounds has been performed at Merck Millipore, Germany.

| **Target** | BMS777607 | LDC047896 |
| --- | --- | --- |
| **ABL** | -74 | -85 |
| **ACK1** | -37 | -31 |
| **ALK** | -3 | -8 |
| **ALK4** | 12 | -5 |
| **ARG** | -61 | -70 |
| **ARK5** | 25 | -19 |
| **ASK1** | 18 | -8 |
| **AURKA** | -55 | -58 |
| **AURKB** | -98 | -95 |
| **AURKC** | -88 | -68 |
| **AXL** | -98 | -71 |
| **BLK** | -24 | -23 |
| **BMX** | -26 | -26 |
| **BRK** | -79 | -20 |
| **BRSK1** | 11 | -11 |
| **BRSK2** | 6 | 4 |
| **BTK(R28H)** | 0 | 2 |
| **CAMKI** | -12 | -11 |
| **CAMKIDELTA** | 9 | -31 |
| **CAMKIIBETA** | 1 | -5 |
| **CAMKIIDELTA** | -8 | -17 |
| **CAMKIIGAMMA** | 8 | 3 |
| **CAMKIV** | 3 | 7 |
| **CDK1/CYCLINB** | 7 | -10 |
| **CDK2/CYCLINA** | -3 | 2 |
| **CDK2/CYCLINE** | 6 | 0 |
| **CDK3/CYCLINE** | -11 | -4 |
| **CDK5/P25** | -5 | 4 |
| **CDK5/P35** | -1 | -21 |
| **CDK6/CYCLIND3** | -9 | -22 |
| **CDK7/CYCLINH/MAT1** | 7 | -7 |
| **CDK9/CYCLINT1** | -2 | -10 |
| **CHK1** | -1 | -7 |
| **CHK2** | -6 | -20 |
| **CHK2(I157T)** | -2 | 2 |
| **CHK2(R145W)** | -6 | -8 |
| **CK1DELTA** | 24 | 8 |
| **CK1GAMMA1** | 34 | -4 |
| **CK1GAMMA2** | -9 | -5 |
| **CK1GAMMA3** | 8 | -7 |
| **CK2** | -12 | -2 |
| **CK2ALPHA2** | 5 | -2 |
| **CKIT** | -4 | -31 |
| **CLK2** | 17 | -11 |
| **CLK3** | 5 | 8 |
| **CRAF** | -88 | -9 |
| **CSK** | -3 | -7 |
| **DAPK1** | 3 | -99 |
| **DAPK2** | 14 | -11 |
| **DCAMKL2** | -6 | -4 |
| **DDR2** | -95 | -4 |
| **DMPK** | 11 | 6 |
| **DRAK1** | 1 | -12 |
| **DYRK2** | 6 | -7 |
| **EEF-2K** | 1 | -7 |
| **EGFR** | -13 | -1 |
| **EPHA1** | -24 | -2 |
| **EPHA2** | -53 | 2 |
| **EPHA3** | -24 | 10 |
| **EPHA4** | -23 | 1 |
| **EPHA5** | -51 | -7 |
| **EPHA7** | -86 | 10 |
| **EPHA8** | -88 | 12 |
| **EPHB1** | -52 | 8 |
| **EPHB2** | -56 | -8 |
| **EPHB3** | -8 | 10 |
| **EPHB4** | -17 | -29 |
| **ERBB4** | -10 | 7 |
| **FAK** | 3 | -6 |
| **FER** | 24 | -8 |
| **FES** | -7 | -11 |
| **FGFR1** | -5 | -81 |
| **FGFR2** | 6 | -79 |
| **FGFR3** | -15 | -57 |
| **FGFR4** | -8 | -5 |
| **FGR** | -59 | -7 |
| **FLT1** | -70 | -93 |
| **FLT3** | -27 | -84 |
| **FLT4** | -99 | -100 |
| **FMS** | -41 | -98 |
| **FYN** | -34 | -4 |
| **GCK** | 2 | -8 |
| **GRK5** | 3 | -6 |
| **GRK6** | -7 | 5 |
| **GRK7** | 8 | 3 |
| **GSK3ALPHA** | -11 | 0 |
| **GSK3BETA** | -25 | -5 |
| **HASPIN** | 26 | 30 |
| **HCK** | -18 | -10 |
| **HCK** | -47 | 25 |
| **HIPK1** | 7 | -4 |
| **HIPK2** | -1 | -3 |
| **HIPK3** | -5 | -6 |
| **IGF-1R** | -8 | -16 |
| **IGF-1R, ACTIVATED** | 7 | -22 |
| **IKKALPHA** | 7 | -2 |
| **IKKBETA** | -16 | 21 |
| **IR** | -26 | -30 |
| **IR, ACTIVATED** | 0 | -13 |
| **IRAK1** | -16 | 12 |
| **IRAK4** | -9 | -5 |
| **IRR** | -14 | 15 |
| **ITK** | 11 | -11 |
| **JAK2** | 16 | -6 |
| **JAK3** | -5 | 5 |
| **JNK1ALPHA1** | -1 | 8 |
| **JNK2ALPHA2** | -2 | 8 |
| **JNK3** | 8 | -6 |
| **KDR** | -90 | -88 |
| **LCK** | -70 | -46 |
| **LCK** | -82 | -77 |
| **LIMK1** | -71 | -48 |
| **LKB1** | 5 | -6 |
| **LOK** | -57 | -18 |
| **LYN** | -46 | -54 |
| **MAPK1** | 11 | -1 |
| **MAPK2** | -2 | -9 |
| **MAPKAP-K2** | -3 | 29 |
| **MAPKAP-K3** | 4 | 5 |
| **MARK1** | 2 | 8 |
| **MEK1** | -2 | -16 |
| **MELK** | -43 | -4 |
| **MER** | -101 | -16 |
| **MET** | -103 | -91 |
| **MINK** | 1 | -16 |
| **MKK6** | -6 | 2 |
| **MKK7BETA** | -1 | -26 |
| **MLCK** | -2 | 0 |
| **MLK1** | -28 | -29 |
| **MNK2** | -89 | -18 |
| **MRCKALPHA** | 9 | 8 |
| **MRCKBETA** | -6 | 0 |
| **MSK1** | -1 | 24 |
| **MSK2** | -11 | -8 |
| **MSSK1** | 18 | -9 |
| **MST1** | 14 | 16 |
| **MST2** | -15 | -23 |
| **MST3** | 5 | 5 |
| **MTOR** | -3 | 0 |
| **MTOR/FKBP12** | 6 | -2 |
| **MUSK** | -85 | -51 |
| **NEK11** | -2 | 18 |
| **NEK2** | -9 | -6 |
| **NEK3** | -5 | 30 |
| **NEK6** | 1 | 1 |
| **NEK7** | 7 | 11 |
| **NLK** | 5 | 6 |
| **P70S6K** | -12 | 14 |
| **PAK2** | -6 | -18 |
| **PAK4** | -2 | -4 |
| **PAK5** | 15 | -8 |
| **PAK6** | -2 | 9 |
| **PAR-1BALPHA** | 5 | 8 |
| **PASK** | -5 | 10 |
| **PDGFRALPHA** | -15 | -52 |
| **PDGFRBETA** | 6 | -73 |
| **PDK1** | 29 | 8 |
| **PEK** | -17 | -26 |
| **PHKGAMMA2** | -1 | -14 |
| **PI3K BETA** | 0 | -3 |
| **PI3K DELTA** | -2 | -3 |
| **PI3K GAMMA** | -3 | -13 |
| **PIM-1** | 10 | 5 |
| **PIM-2** | -93 | -21 |
| **PIM-3** | -5 | 1 |
| **PIP4K2A** | 9 | -4 |
| **PKA** | -50 | 6 |
| **PKBALPHA** | -3 | -2 |
| **PKBBETA** | -18 | -4 |
| **PKBGAMMA** | 3 | 0 |
| **PKCALPHA** | 7 | 5 |
| **PKCBETAI** | -3 | 6 |
| **PKCBII** | 0 | -1 |
| **PKCDELTA** | -2 | -16 |
| **PKCEPSILON** | -6 | -4 |
| **PKCETA** | 22 | 5 |
| **PKCGAMMA** | 1 | -1 |
| **PKCIOTA** | 17 | 4 |
| **PKCMU** | 4 | -16 |
| **PKCZETA** | 1 | -2 |
| **PKD2** | 1 | 3 |
| **PKG1ALPHA** | 13 | -13 |
| **PKG1BETA** | 0 | 5 |
| **PLK1** | 8 | 0 |
| **PLK3** | -2 | 1 |
| **PRAK** | 4 | 2 |
| **PRK2** | -3 | 2 |
| **PRKAA1** | -4 | 9 |
| **PRKAA2** | -2 | -5 |
| **PRKCQ** | -2 | -11 |
| **PRKX** | 6 | 28 |
| **PTK5** | -98 | -44 |
| **PYK2** | -19 | -24 |
| **RET** | -1 | -24 |
| **RIPK2** | 2 | -5 |
| **ROCK-I** | -34 | -15 |
| **ROCK-II** | -29 | -16 |
| **RON** | -100 | -101 |
| **ROS** | -29 | 7 |
| **RSE** | -104 | -26 |
| **RSK1** | 44 | 12 |
| **RSK2** | 3 | 19 |
| **RSK3** | 2 | 7 |
| **RSK4** | -7 | 28 |
| **SAPK2A** | -15 | 12 |
| **SAPK2B** | 2 | 29 |
| **SAPK3** | -1 | 12 |
| **SAPK4** | -2 | -4 |
| **SGK** | 5 | 11 |
| **SGK2** | 0 | -29 |
| **SGK3** | 7 | 11 |
| **SIK** | 13 | -11 |
| **SNK** | 5 | 1 |
| **SRC** | -36 | -6 |
| **SRPK1** | 4 | 0 |
| **SRPK2** | 3 | 17 |
| **STK33** | -5 | -12 |
| **SYK** | -1 | 12 |
| **TAK1** | -30 | -1 |
| **TAO1** | -9 | -7 |
| **TAO2** | -75 | 0 |
| **TAO3** | -5 | -13 |
| **TBK1** | 6 | -2 |
| **TEC ACTIVATED** | 4 | -8 |
| **TGFBR1** | 19 | -23 |
| **TIE2** | -98 | -46 |
| **TLK2** | -1 | 2 |
| **TRKA** | -100 | -57 |
| **TRKB** | -100 | -23 |
| **TSSK1** | -6 | -1 |
| **TSSK2** | -3 | 0 |
| **TXK** | -16 | -2 |
| **ULK2** | 10 | -4 |
| **ULK3** | -17 | 8 |
| **VRK2** | -4 | -3 |
| **WNK2** | -2 | -29 |
| **WNK3** | 12 | -28 |
| **YES** | -7 | -26 |
| **ZAP-70** | -1 | 33 |
| **ZIPK** | -2 | -10 |
